# Children develop robust and sustained cross-reactive spike-specific immune responses to SARS-CoV-2 infection

**DOI:** 10.1038/s41590-021-01089-8

**Published:** 2021-12-22

**Authors:** Alexander C. Dowell, Megan S. Butler, Elizabeth Jinks, Gokhan Tut, Tara Lancaster, Panagiota Sylla, Jusnara Begum, Rachel Bruton, Hayden Pearce, Kriti Verma, Nicola Logan, Grace Tyson, Eliska Spalkova, Sandra Margielewska-Davies, Graham S. Taylor, Eleni Syrimi, Frances Baawuah, Joanne Beckmann, Ifeanyichukwu O. Okike, Shazaad Ahmad, Joanna Garstang, Andrew J. Brent, Bernadette Brent, Georgina Ireland, Felicity Aiano, Zahin Amin-Chowdhury, Samuel Jones, Ray Borrow, Ezra Linley, John Wright, Rafaq Azad, Dagmar Waiblinger, Chris Davis, Emma C. Thomson, Massimo Palmarini, Brian J. Willett, Wendy S. Barclay, John Poh, Gayatri Amirthalingam, Kevin E. Brown, Mary E. Ramsay, Jianmin Zuo, Paul Moss, Shamez Ladhani

**Affiliations:** 1 Institute of Immunology & Immunotherapy, College of Medical and Dental Sciences, University of Birmingham, Birmingham, UK; 2 MRC-University of Glasgow Centre for Virus Research, Glasgow, UK; 3 Public Health England, 61 Colindale Avenue, London, UK; 4 East London NHS Foundation Trust, London, UK; 5 University Hospitals of Derby and Burton NHS Foundation Trust, Derby, UK; 6 Manchester University NHS Foundation Trust, Manchester, UK; 7 Birmingham Community Healthcare NHS Trust, Aston, UK; 8 Institute of Applied Health Research, College of Medical and Dental Sciences, University of Birmingham, Birmingham, UK; 9 Oxford University Hospitals NHS Foundation Trust, Oxford, UK; 10 University of Oxford, Wellington Square, Oxford, UK; 11 Public Health England, Manchester Royal Infirmary, Manchester, UK; 12 Bradford Institute for Health Research, Bradford Teaching Hospitals NHS Foundation Trust, Bradford, UK; 13 Department of Infectious Disease, Imperial College, London, UK; 14 Paediatric Infectious Diseases Research Group, St. George’s University of London, London, UK

**Keywords:** Adaptive immunity, Infectious diseases, Viral infection, SARS-CoV-2

## Abstract

SARS-CoV-2 infection is generally mild or asymptomatic in children but a biological basis for this outcome is unclear. Here we compare antibody and cellular immunity in children (aged 3–11 years) and adults. Antibody responses against spike protein were high in children and seroconversion boosted responses against seasonal Beta-coronaviruses through cross-recognition of the S2 domain. Neutralization of viral variants was comparable between children and adults. Spike-specific T cell responses were more than twice as high in children and were also detected in many seronegative children, indicating pre-existing cross-reactive responses to seasonal coronaviruses. Importantly, children retained antibody and cellular responses 6 months after infection, whereas relative waning occurred in adults. Spike-specific responses were also broadly stable beyond 12 months. Therefore, children generate robust, cross-reactive and sustained immune responses to SARS-CoV-2 with focused specificity for the spike protein. These findings provide insight into the relative clinical protection that occurs in most children and might help to guide the design of pediatric vaccination regimens.

## Abstract (web summary)

SARS-CoV-2 infection is milder in children, but direct comparison with adults is rare. Here the authors show that immune responses are higher in children, retained for 12 months or longer and can neutralize Alpha, Beta and Delta variants.

## Main

The SARS-CoV-2 pandemic has resulted in over 4.2 million deaths so far and the most notable determinant of outcome is age at the time of primary infection^1^. SARS-CoV-2 infection in children is generally asymptomatic or mild and contrasts with high rates of hospitalization and death in older adults^2^. As such, there is interest in understanding the profile of the immune response to SARS-CoV-2 in children. Such studies are limited to date but have reported reduced magnitude of both antibody and cellular responses in comparison to adults and an absence of nucleocapsid-specific antibody responses during or early postinfection^3–6^. One unique feature of SARS-CoV-2 infection in children is the development of a rare complication known as pediatric inflammatory multisystem syndrome temporally associated with SARS-CoV-2 (PIMS-TS), also known as multisystem inflammatory syndrome in children (MIS-C), which shares features with Kawasaki disease and toxic shock syndrome^7,8^. MIS-C develops approximately 2–4 weeks after infection in children with a median age of 9 years^9^. The immunological basis for this condition is unclear but it is characterized by diffuse endothelial involvement and broad autoantibody production^10^.

One potential determinant of differential immune responses to SARS-CoV-2 across the life course may be the timing of exposure to the four additional endemic human coronaviruses (hCoVs). These comprise the Beta-coronaviruses OC43 and HKU-1, which have 38% and 35% amino acid homology with SARS-CoV-2, and the more distantly related Alpha-coronaviruses NL63 and 229E, each with around 31% homology^11^. These coronaviruses cause frequent mild childhood infections and antibody seroconversion occurs typically before the age of 5 years. Infection with one of the Alpha- or Beta-coronaviruses provides short-term immunity against reinfection from coronaviruses and represents transient cross-reactive immunity within the subtypes^12–14^. As such, recent hCoV infection might presensitize children against SARS-CoV-2 infection and may explain cross-reactive SARS-CoV-2-neutralizing antibodies in some seronegative children^15^. Immune responses against hCoV are retained throughout life but do not provide sterilizing immunity^13^. Consequently, recurrent infections are common, generating concern that a similar pattern will be observed after SARS-CoV-2 infection.

COVID-19 vaccines are now being administered widely to adult populations and are also being delivered to children in some countries. Therefore, it is imperative to understand the profile of SARS-CoV-2-specific immune responses in children after natural infection to inform vaccination strategy. In this study, we provide a comprehensive characterization of the convalescent humoral and cellular immune response in a cohort of 91 primary school-aged children compared with 154 adults taking part in the COVID-19 surveillance in school KIDs (sKIDs) study^16^. We demonstrate a markedly different profile of immune response after SARS-CoV-2 infection in children compared to adults. These findings have potential implications for understanding protective or pathological immune responses to infection in children and might help to guide and interpret COVID-19 vaccination regimens for children.

## Results

### Children develop robust antibody responses to SARS-CoV-2

**Extended Data Fig. 1:**
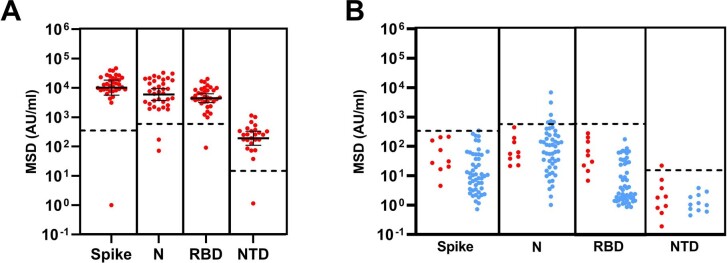
MSD assay specificity and sensitivity. (**a**) MSD results using convalescent plasma samples from 35 children with PCR-confirmed SARS-CoV-2 infection. Bars indicate Geometric Mean±95%CI. (**b**) Assay cut-offs were tested using plasma samples from nine children (red) and 50 adults (blue) taken prior to COVID-19. Dotted lines indicate the cut-off used. Source data

Blood samples were obtained from 91 children and 154 adults, including 35 children and 81 adults known to be seropositive in previous rounds of testing. All infections were asymptomatic or mild and no staff or students in the cohort required medical care or hospitalization. The median age of children was 7 years (range 3–11) while that of adults was 41 years (range 20–71). The SARS-CoV-2 antibody profile was assessed using the Meso Scale Discovery (MSD) V-PLEX serology platform to determine serological responses against spike, receptor binding domain (RBD), N-terminal domain (NTD) and nucleocapsid (N). In total, 47% of children and 59% of adults were seropositive (Supplementary Table 1). To ensure the sensitivity of our assays, we obtained convalescent plasma samples from 35 children with PCR-confirmed SARS-CoV-2. Thirty-four were seropositive in the assay while 1 donor mounted no detectable antibody response to any antigen tested. Prepandemic plasma samples from 9 children and 50 adults all gave negative results and demonstrated the specificity of the assay (Extended Data Fig. 1).

**Fig. 1:**
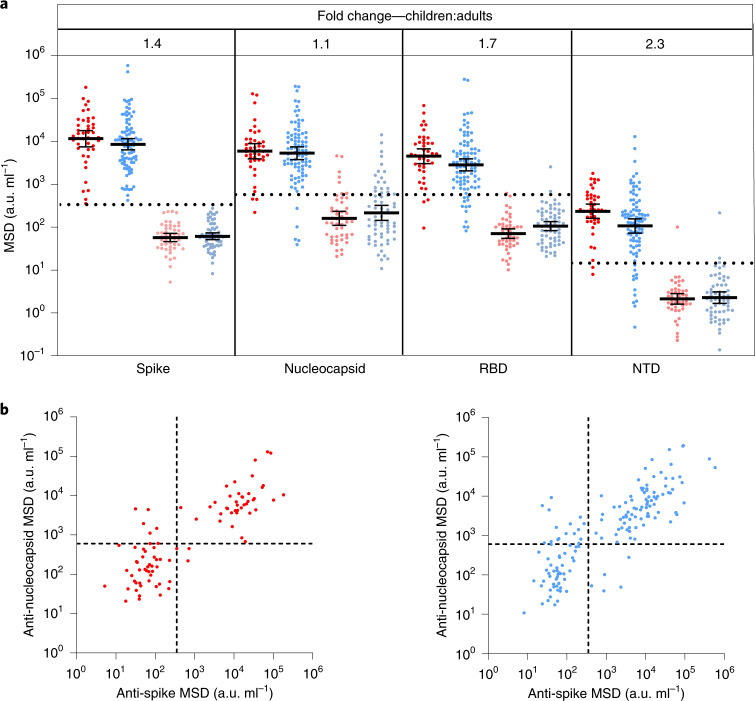
Children and adults develop coordinated antibody responses to SARS-CoV-2. **a**, SARS-CoV-2 antibody levels measured by MSD assay in children (*n* = 91) and adults (*n* = 154). Serostatus was assigned based on spike serology and used to divide the cohorts into seropositive (red/blue) and seronegative (light red/light blue) (seropositive/negative children *n* = 43/48, adults *n* = 91/63, respectively). The dotted lines represent cutoff values for serostatus. Fold change indicates the difference between the GMTs in seropositive children and adults. The bars indicate the geometric mean with 95% confidence interval (CI). **b**, The level of the spike- and nucleocapsid-specific antibody response was correlated within individual donors and revealed a coordinated response to both proteins. a.u., arbitrary unit. Source data

Seropositive children and adults demonstrated broadly similar antibody responses against viral proteins. However, geometric mean antibody titers against all four regions were higher in children, most notably against the NTD and RBD domains, which showed 2.3-fold and 1.7-fold increases, respectively, although these did not reach statistical significance (Fig. 1a). In contrast to previous reports^3^, we also observed antibody responses against nucleoprotein, with a 1.3-fold increased antibody titer compared to adults (Fig. 1a,b).

### SARS-CoV-2 infection boosts hCoV binding antibodies in children

Pre-existing immune responses against seasonal coronaviruses might act to modulate clinical outcome after primary SARS-CoV-2 infection and cross-reactive neutralizing antibodies have been reported in SARS-CoV-2-seronegative children^15^. Consequently, we compared antibody levels against the four hCoVs in SARS-CoV-2 in seronegative and seropositive children and adults.

**Fig. 2:**
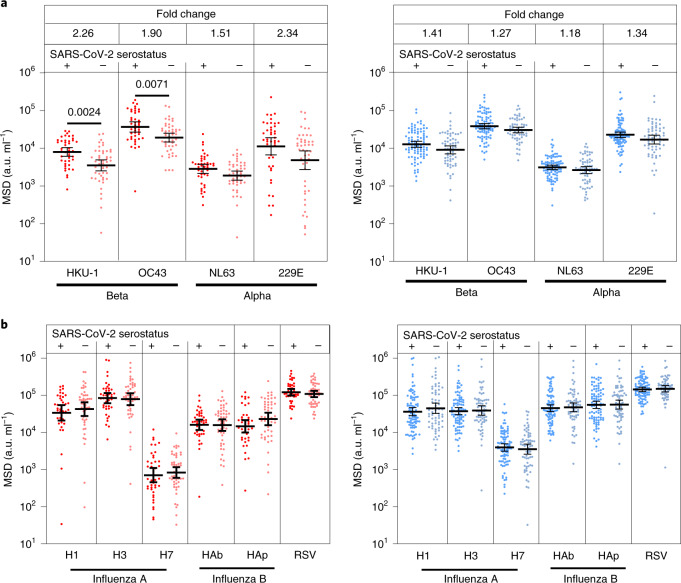
Antibody responses to hCoVs are back-boosted by SARS-CoV-2 in children. **a**,**b**, Antibody titers to the seasonal hCoV coronaviruses (**a**) and other respiratory viruses (**b**) in children (red) and adults (blue) based on SARS-CoV-2 serostatus (dark, seropositive, light, seronegative; seropositive/negative children *n* = 43/48, adults *n* = 91/63, respectively). Fold change indicates the difference between the GMTs in seropositive children and adults. The bars indicate the geometric mean with the 95% CI. Only significant differences are shown. Brown–Forsythe and Welch’s ANOVA with Dunnett’s T3 multiple comparison tests were used. Source data

A 1.2–1.4-fold increase in the titer against hCoV was evident in SARS-CoV-2 seropositive adults compared to the seronegative group. In contrast, antibody levels against all 4 viruses were boosted markedly in SARS-CoV-2 seropositive children, with 2.3, 1.9, 1.5 and 2.1-fold higher antibody levels compared to the seronegative group. These were significant for OC43 and HKU-1 (*P* = 0.0071 and *P* = 0.0024 Brown–Forsythe and Welch’s analysis of variance (ANOVA), with Dunnett’s T3 multiple comparison test) (Fig. 2a). Notably, the level of hCoV-specific antibodies in seropositive children was comparable to adults, whereas seronegative children possessed lower responses than adults (Supplementary Table 1).

To assess if this effect was specific to hCoV or represented a more general effect of SARS-CoV-2 infection on antibody responses against heterologous infection, we also examined antibody titers against influenza subtypes and respiratory syncytial virus in relation to SARS-CoV-2 serostatus. No change in antibody titer against these viruses was seen in either children or adults (Fig. 2b). These data show that SARS-CoV-2 infection in children specifically boosts humoral responses against hCoV.

### SARS-CoV-2 antibodies in children cross-react with Beta-hCoV

Given the increase in hCoV-specific antibody titers after SARS-CoV-2 infection in children, we next assessed to what extent this was cross-reactive against SARS-CoV-2 or could represent an hCoV-specific response. As such, recombinant S1 or S2 domain protein from SARS-CoV-2 was used to preabsorb plasma samples before assessment of antibody levels to both SARS-CoV-2 and the four hCoV subtypes.

**Fig. 3:**
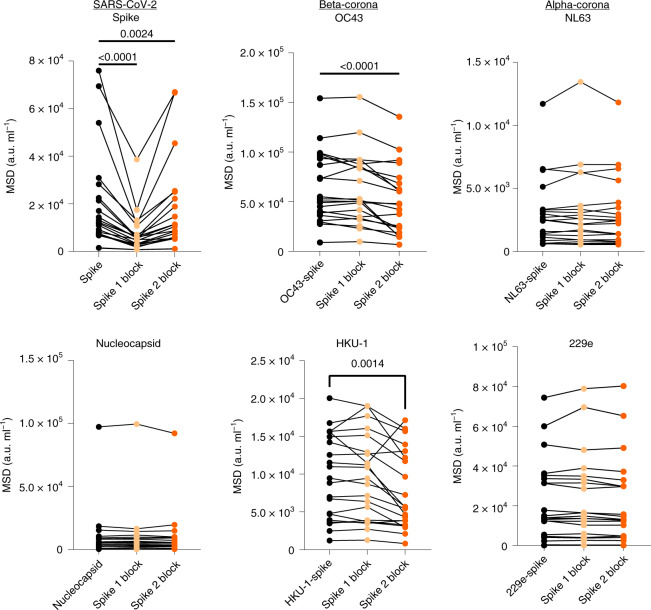
SARS-CoV-2 S2 domain antibodies cross-react with hCoV. Plasma from SARS-CoV-2 seropositive children (*n* = 21) was assessed for binding to the spike protein of the 4 hCoVs or the spike or nucleocapsid regions of SARS-CoV-2. Plasma was either applied neat (control) or after preabsorption with either recombinant spike S1 domain (spike 1 block) or spike S2 domain (spike 2 block). S1 preabsorption markedly reduced binding to SARS-CoV-2 spike with no effect on hCoV, while S2 preabsorption reduced binding to OC43 and HKU-1. One-way repeated measures ANOVA with Holm–Sidak’s multiple comparison test or Friedman test with Dunn’s multiple comparisons test were used as appropriate. Source data

As expected, preabsorption with both the S1 and S2 domains markedly reduced antibody titers against total spike (*P* < 0.0001 and *P* = 0.0024, respectively, Friedman test with Dunn’s multiple comparisons test). The S1 domain, but not the S2 domain, absorbed RBD- and NTD-specific antibodies against SARS-CoV-2 while no influence was observed in relation to nucleocapsid-specific binding for either domain (Fig. 3). Of note, the S1 domain did not reduce antibody binding to any of the four hCoV subtypes, indicating little evidence for cross-reactive antibodies against this domain. The S2 domain, however, selectively reduced antibody binding to the two hCoV Beta-coronaviruses OC43 and HKU-1 (*P* < 0.0001 and *P* = 0.0014, respectively by one-way repeated measures ANOVA with Holm–Sidak’s multiple comparison test). No such effect was observed in relation to binding to the Alpha-coronaviruses NL63 and 229e (Fig. 3).

These data show that the S1 region is the immunodominant target of antibody responses in children. However, antibodies that are cross-reactive against Beta-coronavirus are largely specific for the S2 domain and contribute to the higher SARS-CoV-2-specific titer in children.

### Children develop robust cellular immune responses to spike protein

We next assessed the magnitude and profile of the cellular immune response against SARS-CoV-2 in children and adults. Enzyme-linked immunosorbent spot (ELISpot) analysis against overlapping peptide pools from spike and a combination of nucleocapsid and membrane and envelope (nucleoprotein and viral membrane (N/M)) was performed on samples from 57 children and 93 adults, including 37 and 64 respectively who were seropositive.

As expected, ELISpot responses were common in SARS-CoV-2 seropositive donors with 89% (33 out of 37) of seropositive children and 80% (51 out of 64) of seropositive adults showing a positive ELISpot response to spike and/or the N/M pool.

**Fig. 4:**
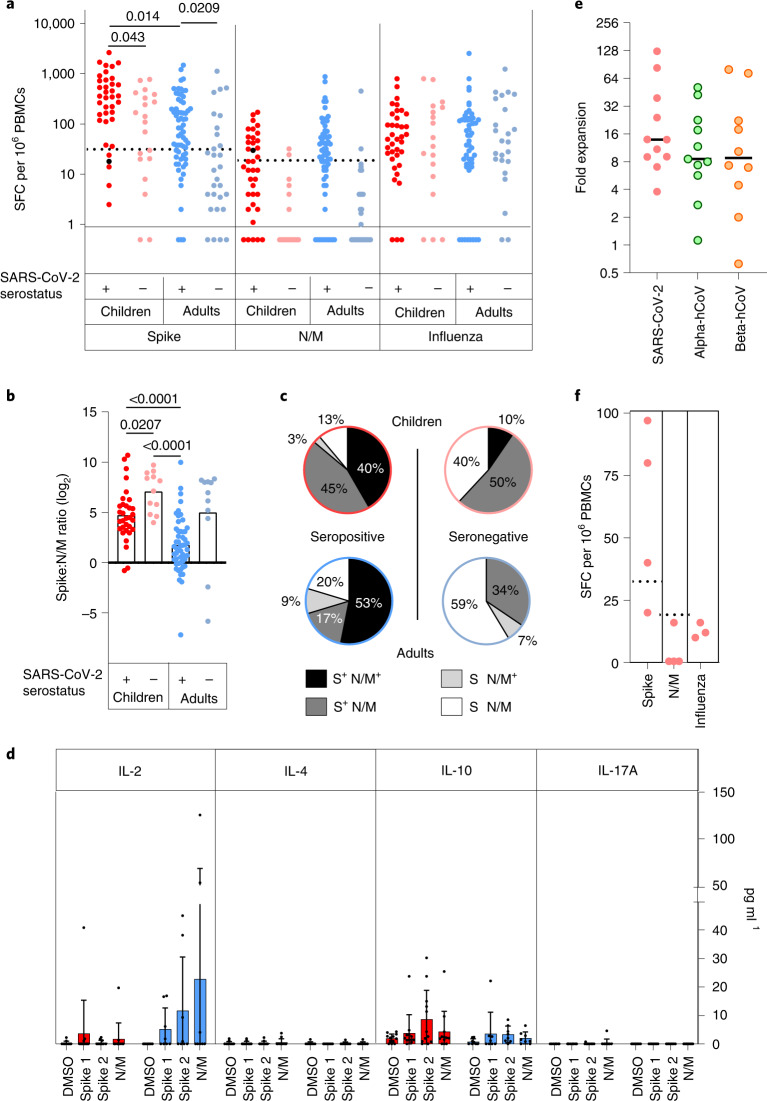
Spike-specific T cell responses in SARS-CoV-2 seropositive and seronegative children. **a**, SARS-CoV-2-specific T cell responses in children (*n* = 57, red) and adults (*n* = 83, blue) based on SARS-CoV-2 serostatus (dark; seropositive, light; seronegative). SARS-CoV-2 serostatus was 37/20 seropositive or negative in children and 64/29 seropositive or negative in adults, respectively. The assay used IFN-γ ELISpot using pepmixes containing overlapping peptides to spike, N/M or influenza and is shown in relation to serostatus. **b**, The magnitude of the spike-specific cellular response was compared to that against N/M and displayed as a ratio in seropositive and seronegative adults and children, as indicated. The bars indicate the mean. Brown–Forsythe and Welch’s ANOVA with Dunnett’s T3 multiple comparisons tests were used. **c**, Proportions of individuals within each cohort who demonstrated a cellular response to S or N/M peptides from SARS-CoV-2. **d**, Cytokine concentration within supernatants from the ELISpot cultures (*n* = 12 children, red; *n* = 8 adults, blue). The bars indicate the mean ± s.d. **e**, hCoV-specific cellular responses showed equivalent expansion after stimulation of PBMCs from SARS-CoV-2 seronegative children with the SARS-CoV-2 S2 domain pepmix (*n* = 11). Cultures were stimulated for 9 d and then assessed by IFN-γ ELISpot to the pepmix of the S2 domain from SARS-CoV-2 or the Alpha (OC43 and HKU-1) or Beta (NL63 and 229E) hCoV. Expansion is shown relative to unstimulated control cultures. The lines indicate the median. **f**, SARS-CoV-2-specific T cell response in PBMC samples taken from children before the COVID-19 pandemic (*n* = 4). Dotted lines in **a** and **f** indicate pre-defined positive thresholds. Source data

The magnitude of the cellular response against spike was 2.1-fold higher in children, with median values of 533 spots per million compared to 195 in adults (*P* = 0.0003, Brown–Forsythe and Welch’s ANOVA with Dunnett’s T3 multiple comparisons test) (Fig. 4a). Cellular responses against the N/M pool were relatively lower in children compared to spike such that the S:N/M ratio was markedly elevated in children at 4.7 compared to 1.8 in adults (*P* = 0.0007, Brown–Forsythe and Welch’s ANOVA with Dunnett’s T3 multiple comparisons test) (Fig. 4b). Eighty-six percent of children showed a positive response to spike while only 43% responded to the N/M pool. Within adults these values were 70% and 63%, respectively (Fig. 4c).

**Extended Data Fig. 2:**
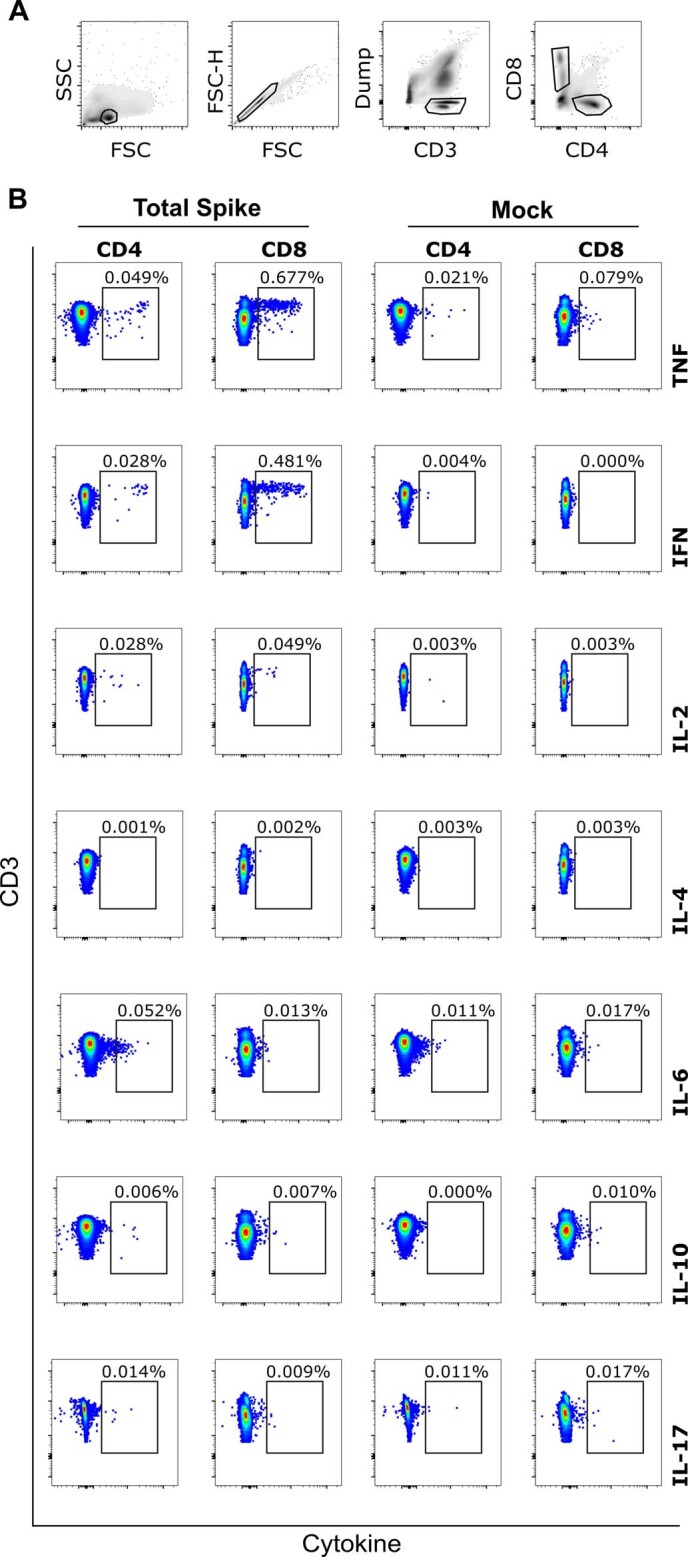
CD8 + T cells with a IL-2^−^TNF^+^IFN-γ^+^ phenotype dominate the spike-specific T cell response in children. PBMC from SARS-CoV-2 seropositive children were stimulated for 6hrs in the presence of spike peptide pool and then cytokine production analysed by flow cytometry by intracellular cytokine staining (ICS). (**a**) Gating strategy for analysis. (**b**) Representative example of ICS staining from one child at six months post SARS-CoV-2 infection showing TNF + IFN-γ + CD8 + T cell response representing 0.48% of the global CD8 + T cell repertoire. Source data

The supernatant from ELISpot was analyzed using a multi-analyte bead assay to compare the profile of cytokine production by SARS-CoV-2-specific T cells from children and adults. Samples from adult donors showed a marked interleukin-2 (IL-2) response with lower levels of interleukin-10 (IL-10) production. In contrast, IL-2 levels in samples from children were very low (Fig. 4d), indicating differential functional response compared to adults. Indeed, analysis of three children by flow cytometry indicated that CD8^+^IL-2-TNF^+^IFN-γ^+^ T cells constituted the bulk of the spike-specific T cell response in children (Extended Data Fig. 2).

Notably, cellular responses were also observed in 60% (12 out of 20) of seronegative children, all of whom were seronegative by 3 different serology platforms. Cellular responses of variable but lower magnitude were also present in 34% (10 out 29) of seronegative adults (Fig. 4a). Two children and one adult classed as seronegative had anti-nucleocapsid antibodies (Supplementary Table 2) but this was insufficient to provide definitive serostatus. These cellular responses in seronegative donors were markedly spike-specific, with elevated S:N/M ratios of 7.1 and 5 in children and adults, respectively (Fig. 4b), indicating pre-existing cross-reactive immunity. Indeed seronegative children (7 out of 12) and seronegative adults (6 out of 10) with a positive ELISpot demonstrated high antibody levels to 1 or more hCoVs (Supplementary Table 2), potentially indicating recent hCoV infection.

To examine the presence of cross-reactive T cells in seronegative children, we hypothesized that expansion of T cells in response to SARS-CoV-2 would be associated with an increase in hCoV-reactive responses. As such we first stimulated cells from SARS-CoV-2 seronegative donors with SARS-CoV-2 peptides and then assessed the response to peptides from SARS-CoV-2 and also peptide pools from the Alpha and Beta hCoVs. Markedly increased cellular responses to both Alpha and Beta hCoVs were seen after culture (8.6-fold and 8.9-fold, respectively) (Fig. 4e), indicating SARS-CoV-2-driven expansion of broadly cross-reactive T cells. Finally, to definitively assess the presence of cross-reactive T cells in children, we obtained prepandemic PBMCs from children and observed that 50% of these had notable responses to spike by ELISpot but lacked cellular responses against N/M peptides (Fig. 4f). Matched plasma samples, available for three donors, showed that antibody responses against HKU-1 and 229E were twofold and fivefold greater in a donor with high cellular responses compared to two children who lacked cellular responses.

Overall these data show that cross-reactive coronavirus-specific T cell responses are present within a high proportion of children.

### Children maintain SARS-CoV-2-specific immune responses for 12 months

**Fig. 5:**
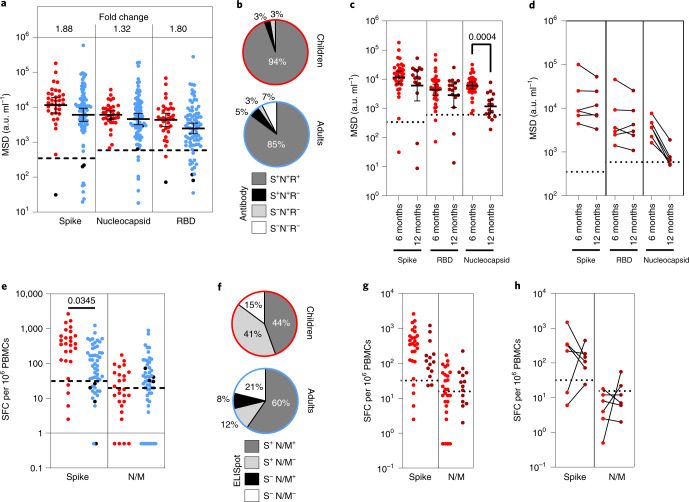
Immune responses are maintained in children at least six months after infection. **a**, Antibody responses in children (*n* = 35, red) and adults (*n* = 81, blue) who were seropositive at first testing and therefore at least 6 months post-primary infection. The bars indicate the GMT ± 95% CI. The dotted lines indicate seropositive cutoffs. Fold change indicates increment in children’s GMT compared to adults. The black dots indicate individuals who were seronegative for spike but retained a nucleocapsid-specific antibody response. **b**, Proportion of individuals retaining antibody responses to spike, nucleocapsid or RBD at ≥6 months. **c**, Antibody binding to spike, RBD or nucleocapsid in children ≥6 months (*n* = 35, red) or ≥12 months (*n* = 16, dark red) after infection. The bars indicate the GMT ± 95% CI. The dotted lines indicate seropositive cutoffs. A one-way Kruskal–Wallis with Dunn’s multiple comparisons test was used. **d**, Paired antibody levels for children (*n* = 6) at ≥6 and ≥12 months post-primary infection. The dotted lines indicate positive cutoffs. **e**, Spike-specific IFN-γ ELISpot in children (*n* = 27, red) and adults (*n* = 52, blue). The black dots indicate individuals who lacked a spike-specific response but retained a nucleocapsid-specific response. Brown–Forsythe and Welch’s ANOVA with Dunnett’s T3 multiple comparisons tests were used. SFC, spot-forming cell. **f**, Proportion of each cohort scored as responding to SARS-CoV-2 by ELISpot. **g**, Spike-specific IFN-γ ELISpot in children ≥6 months (*n* = 27, red) or ≥12 months (*n* = 14, dark red) after infection. The dotted lines indicate seropositive cutoffs. **h**, Paired ELISpot results for children at ≥6 and ≥12 months post-primary infection (*n* = 6). The dotted lines indicate seropositive cutoffs. Source data

We next assessed the longevity of immune responses within a subgroup of 35 children and 81 adults who had seroconverted at least 6 months before the analysis. All children retained humoral immunity while 7% (6/81) of previously seropositive adults failed to show antibody responses. Children also maintained higher antibody titers against spike and RBD, which were 1.8-fold higher than adults (Fig. 5a,b).

Samples were also obtained from 16 children who had seroconverted at least 12 months before the analysis. Antibody levels to spike and RBD were retained at a similar, although slightly reduced, level to those seen at 6 months while nucleocapsid-specific antibody levels were reduced (*P* = 0.0004) (Fig. 5c). Two of these 16 children (12.5%) had spike-specific antibody levels below the threshold while 4 children (25%) showed similar loss of nucleocapsid-specific antibodies, 1 of whom also lost the spike-specific response. Six of these donors had been analyzed previously at 6 months; matched individual comparisons revealed stable spike-specific antibody levels between 6 and 12 months postinfection whereas nucleocapsid-specific antibodies showed substantial waning, with 2 donors dropping below the cutoff (Fig. 5d).

Cellular immune responses were detectable in 84% of children and 79% of adults at least 6 months after infection. The magnitude of the spike-specific response was higher in children than in adults (*P* = 0.032) whereas responses to the N/M pool were seen in only 31% of children compared to 68% of adults (Fig. 5e,f). Fifteen children who had seroconverted over 12 months previously were also assessed by ELISpot. Compared to the cohort analysis at 6 months, T cell responses to spike were retained but somewhat reduced, while nucleocapsid-specific responses, although of lower magnitude, were retained at a similar level (Fig. 5g). Matched samples at 6 and 12 months were available for 5 of these children and were stable. These data show that children broadly retained both antibody and cellular responses for extended periods after primary infection (Fig. 5h).

### Enhanced binding but equal neutralization of variants of concern in children

SARS-CoV-2 variants of concern (VOC) may be able to partially escape immunity generated by previous infection or vaccination^17,18^. Given the development and maintenance of cross-reactive high-level antibody responses in children, we assessed their relative recognition of spike protein from VOC. Plasma samples from 19 children and 18 adults at >6 months after primary infection were tested for binding to spike and RBD from Alpha (B.1.1.7), Beta (B.1.351) and Gamma (P.1) variants compared to the original Wuhan genotype, which was used in previous assays.

**Fig. 6:**
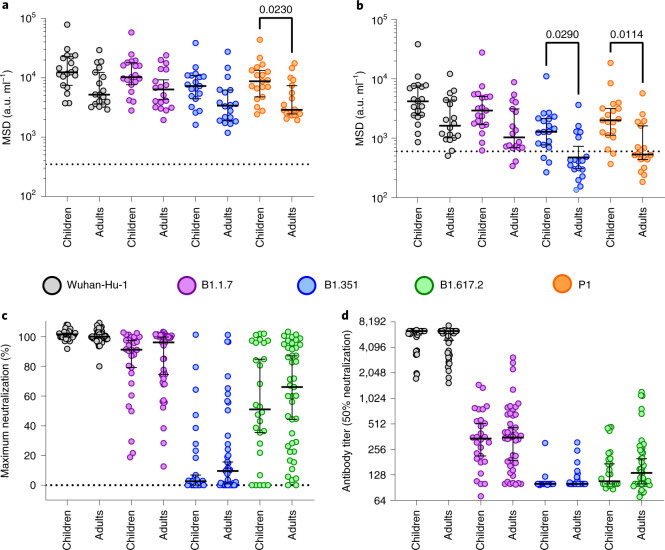
Superior antibody binding of SARS-CoV-2 variants in children and comparable neutralization. **a**,**b**, Antibody binding to spike (**a**) and RBD proteins (**b**) from SARS-CoV-2 variants using plasma from children (*n* = 19) or adults (*n* = 18). The bars indicate the geometric mean ± 95% CI. Kruskal–Wallis with Dunn’s multiple comparisons tests were used. **c**,**d**, Live virus neutralization assays on SARS-CoV-2 variants displayed as maximal neutralization of infection (**c**) and titer at 50% neutralization (**d**) using plasma from children (*n* = 28) or adults (*n* = 43). The bars indicate the median ± 95% CI. Source data

**Extended Data Fig. 3:**
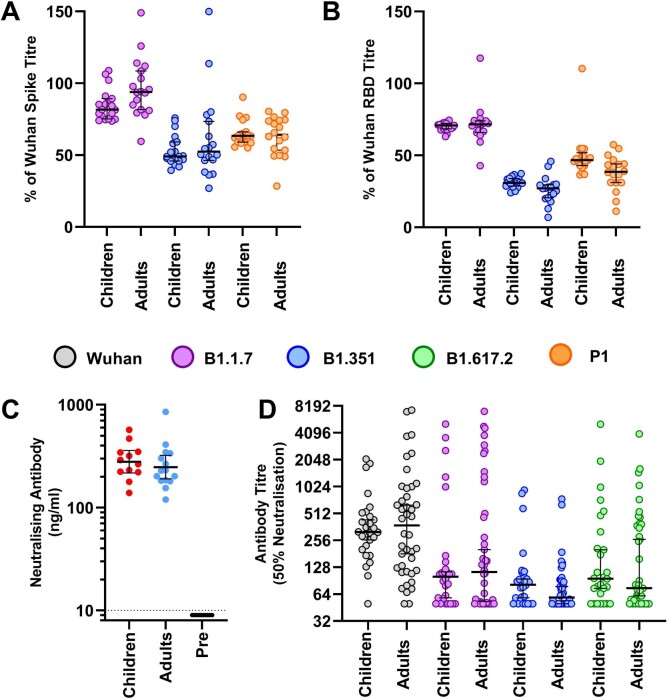
Antibody binding, RBD-ACE2 inhibition and pseudo-neutralisation of SARS-CoV-2 VOC in plasma from children or adults. (**a**) Antibody titres normalised to the Wuhan sequence as determined by MSD to total spike and (**b**) RBD from VOC as indicated in children (n = 19) and adults (n = 18) ≥6 months post primary infection. Bars indicate Median±95%CI. (**c**) Antibody inhibition in an RBD-ACE2 competitive binging assay using plasma ≥6 months post infection in children (n = 12) and adults (n = 15). Results from pre-pandemic adult samples are also shown (n = 10). Bars indicate geometric mean ±95% CI. (**d**) Results from pseudo-virus neutralisation assays displayed as titre at 50% neutralisation from children (n = 28) and adults (n = 43) ≥6 months post primary infection. Bars indicate Median±95%CI. Source data

As described above, children, compared to adults, maintained higher levels of antibody binding to Wuhan spike (Figs. 5a and 6a) and this was also observed in binding to spike from the 3 VOC with 1.7, 1.8 and 2.1-fold higher geometric mean titers (GMTs) against Alpha, Beta and Gamma variants, respectively (Fig. 6a). Children also demonstrated higher binding to the RBD region of the 3 VOC, compared to adults, with 2.1, 1.8 and 2.9-fold higher GMTs, respectively (*P* = 0.029 and *P* = 0.0114 against Beta and Gamma, respectively; Kruskal–Wallis test with Dunn’s multiple comparison test). Applying the threshold determined in earlier assays, 16 out of 19 (84%) children retained seropositive status to both Beta and Gamma compared to only 5 out of 18 (28%) and 8 out of 18 (44%) of adults, respectively (Fig. 6b). Similar ratios of relative binding or inhibition of spike-angiotensin-converting enzyme 2 (ACE2) engagement with spike from Wuhan or VOC were seen in children and adults (Extended Data Fig. 3a,b and Supplementary Table 3), indicating that enhanced antibody binding to VOC is a function of overall quantitively superior antibody responses in children.

We next assessed the ability of sera from children and adults to neutralize infection by live virus. Serum from adults showed markedly reduced capacity to neutralize the VOC and this was particularly noteworthy for B.1.351 (Fig. 6c,d). This pattern was also seen in children and no difference was seen in either maximal or 50% neutralization titer between adults and children. A similar profile was seen with a pseudotype-based neutralization assay (Extended Data Fig. 3d).

These data show that children developed higher antibody binding to SARS-CoV-2 VOC after natural infection compared to adults but displayed similar neutralizing ability.

## Discussion

Age is the primary determinant of the clinical severity of SARS-CoV-2 infection and a life course assessment of virus-specific immunity is essential to understand disease pathogenesis and design vaccine strategies in children. Our detailed analysis of adaptive immune memory identifies a number of important features in young children. A key finding was that the magnitude of the adaptive immune response to SARS-CoV-2 is higher in children compared to adults. This is somewhat different to previous reports that showed lower T cell responses in children^6^. This may reflect differences in assay systems since we used separate spike and N/M peptide pools to demonstrate the heightened spike-specific response. It has also been reported that children do not mount effective antibody responses against nucleocapsid in the early postinfection period^3,5^. Using the well-validated MSD system, we observed nucleocapsid-specific antibody responses in children but it was noteworthy that immune responses were much more focused against spike^19^. Nucleocapsid is an abundant protein within the SARS-CoV-2 virion and it is possible that the magnitude of the N-specific response is a reflection of peak or aggregate viral load. The levels of virus within the upper airways at the time of primary infection are equivalent in children and adults^20^ but relative changes over the course of infection are not known. Enhanced innate immune responses in children may also play an important role in limiting systemic replication and may explain the higher rates of asymptomatic and mild illness in children compared to adults^21^. Antibody levels generally correlated with disease severity but none of the children or adults in this study suffered from severe disease or needed hospital admission.

**Fig. 7:**
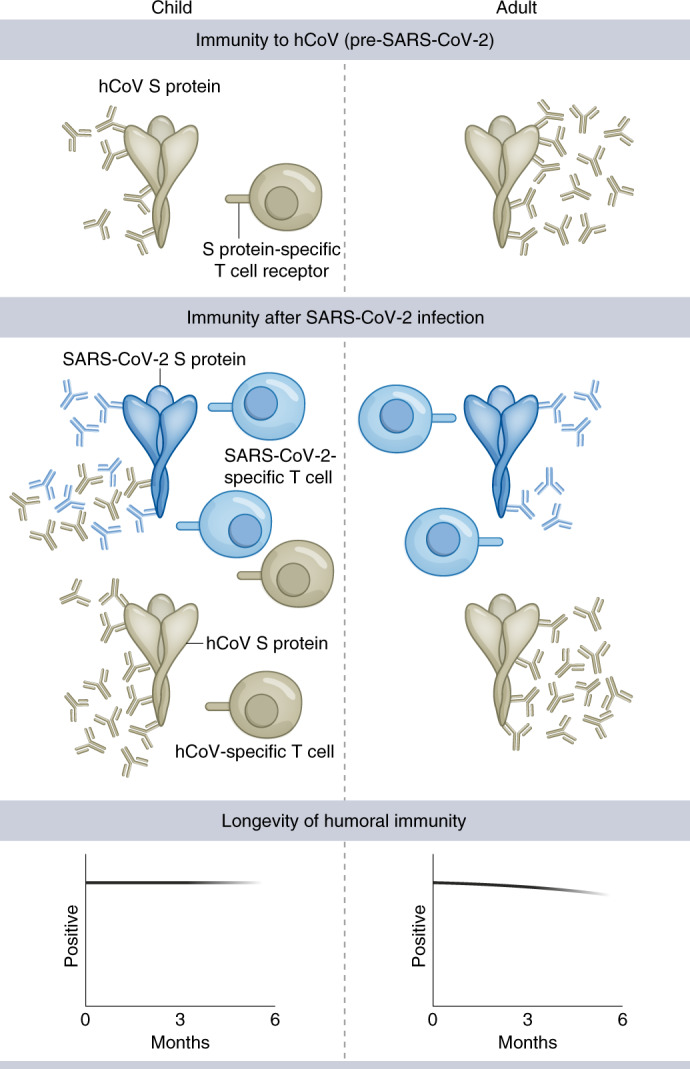
Model of adaptive immunity to coronaviruses in children and adults. Children develop hCoV spike S2-specific antibodies and cellular responses that can cross-react with SARS-CoV-2. Robust S1-specific adaptive responses develop after SARS-CoV-2 infection. Cross-reactive S2-specific responses probably contribute to immune control in children.

One striking feature was that SARS-CoV-2 infection in children doubled antibody titers against all four Alpha and Beta hCoV subtypes. This pattern was not seen in adults where increased humoral responses were modest^22,23^. Of note, increased antibody titers against hCoVs have also been observed after SARS-CoV-1 infection^24^. Using protein domain preabsorption, we found that most of this response resulted from SARS-CoV-2-specific humoral responses that cross-reacted with the S2 domain of the two more closely related Beta-coronaviruses. The S2 domain is more highly conserved between hCoVs than S1 and this pattern is compatible with preferential targeting of structurally conserved epitopes by hCoV-specific antibodies in children^25^ with the potential for neutralizing activity against SARS-CoV-2 (refs. ^15,26^). However, SARS-CoV-2 infection in children also boosted hCoV-specific antibody responses that were not directly cross-reactive, as demonstrated by increased titers against Alpha-coronaviruses that could not be preabsorbed (Fig. 7). This may potentially reflect weakly cross-reactive B cell clones, potentially activated through T cell cross-recognition and is reminiscent of antibody boosting against H3 hemagglutinin after H1N1 infection in children with previous H3N2 infection^27^. We found that titers of hCoV-specific antibodies were lower in seronegative children compared to adults but these are likely to increase after primary infection during the late teenage years^13,25^; it appears that repeated infections may hone S1 domain-specific responses and lead to loss of cross-reactive S2-specific responses in adulthood^15^. Our data show that SARS-CoV-2 infection acts to fill the CoV-specific antibody space, producing antibodies against all coronaviruses and potentially supporting immunity in later life.

The development of antibodies against Alpha-coronaviruses that are not absorbed by the S2 domain of SARS-CoV-2 suggests that there may be a lower affinity threshold for boosting related but heterologous immune responses in children, which might potentially represent an evolutionary adaptation to expand the memory pool early in life. It is interesting to speculate whether this may provide insight into the pathophysiology of PIMS-TS/MIS-C, where B cell activation drives a hyperinflammatory syndrome. Lack of pre-existing immunity to hCoV is a risk factor for PIMS-TS/MIS-C and may indicate a protective influence from a previous cross-reactive memory B cell pool^8^. Furthermore, the pathogenic antibodies associated with this condition are enriched for antibodies against heterologous pathogens and inflammation may result from Fc receptor-mediated monocyte activation^28^.

This profile of an enhanced and cross-reactive humoral immune response in children was also apparent in the analysis of the cellular response to SARS-CoV-2. We found that the virus-specific T cell response was higher in children compared to adults and this mirrored the humoral response in that responses against the spike protein were markedly increased compared to nucleocapsid and envelope proteins. Virus-specific T cell responses in children also showed a differential cytokine response with markedly reduced production of IL-2. This may suggest a more highly differentiated profile in children compared to adults, which is in line with the enhanced magnitude of the response. Indeed, the spike-specific CD8^+^ T cell pool in children at 6 months after primary infection was dominated by an IL-2^−^IFN-γ^+^TNF^+^ phenotype. As such, further long-term characterization of the T cell response in children, and the potential mechanisms that may act to drive T cell activation, are required.

We also observed that cross-reactive CoV-specific T cells are present in many children, both before and after SARS-CoV-2 infection. SARS-CoV-2-specific T cell responses were detectable in more than half of seronegative children, including samples taken prepandemic, and are likely to represent hCoV-specific T cell responses that cross-react against SARS-CoV-2 peptides^29,30^. Of note, young adults were recently reported to have higher T cell responses to hCoV than older people and these can cross-react with SARS-CoV-2 (ref. ^31^). SARS-CoV-2-reactive T cells were also seen in some seronegative adults and it is also possible that some of these responses represent genuine SARS-CoV-2-specific T cells that have been generated after virus exposure in the absence of antibody seroconversion. This pattern has been reported in health care workers with high levels of viral exposure^32^ and it is possible that such conditions are also seen in primary schools where enforcement of social distancing is challenging.

It is tempting to speculate that this profile of cross-reactive antibody and cellular responses in children contributes to the excellent clinical outcomes in this group. In this regard, it would be interesting to assess matched pediatric samples from before and after SARS-CoV-2 infection to determine the relative contribution of cross-reactive hCoV-specific clones to the protective adaptive immune response.

Many participants were seropositive 6 months before the analysis and our data extend previous findings in adults^33,34^ to show sustained immunity over this time period. Moreover, we also found that children maintained higher antibody and cellular immune responses at this time point compared to adults with no loss of humoral response compared to loss in 7% of adults. There is increasing confidence in the relative stability of SARS-CoV-2-specific memory B cell and antibody responses but studies of antibody waning after natural infection are now difficult to perform in adults due to the widespread adoption of COVID-19 vaccines. Spike-specific antibody responses were also largely maintained in children at 12 months, whereas responses against nucleocapsid showed waning. T cell responses were broadly stable; it is important to consider that ELISpot analysis will only detect effector populations and a long-term memory subset is likely to have been established by this point. We could not compare 12-month values to the adult population because this latter group had undergone COVID-19 vaccination before this time point.

We also found that children possessed enhanced binding of antibodies to spike and RBD from viral VOC^18^. However, levels of neutralizing antibodies in live and pseudo-virus neutralization assays were comparable between adults and children. This suggests that children and adults develop comparable neutralizing antibody responses against the spike S1 domain but the increased antibody response in children results from antibodies targeting non-neutralizing epitopes within S1 and S2. These antibodies could still have important effector potential through mechanisms such as antibody-directed cell cytotoxicity; further studies to examine the specificity and function of the SARS-CoV-2 B cell repertoire in children after natural infection or vaccination would be of value. In the light of the concern that SARS-CoV-2 will become an endemic infection^35^, these findings augur well for immunity generated in childhood providing robust and sustained protection, including to emerging VOC.

In conclusion, we showed that children display a characteristically robust and sustained adaptive immune response against SARS-CoV-2 with substantial cross-reactivity against other hCoVs. This is likely to contribute to the relative clinical protection in this age group but these findings may also provide insight into the characteristic immunopathology that may develop. Furthermore, they will help to guide the introduction and interpretation of vaccine deployment in the pediatric population.

## Methods

### Sample collection

Public Health England (PHE) initiated prospective SARS-CoV-2 surveillance in primary schools across the UK after they reopened following the easing of national lockdown in June 2020. The protocol for sKIDs is available online (https://www.gov.uk/guidance/covid-19-paediatric-surveillance)^16^. Surveillance consisted of 2 arms, one involving weekly swabbing of primary school students and staff for SARS-CoV-2 infection (from June to mid-July 2020) and the other comprising swabbing and blood sampling taken in 3 rounds: beginning (1–19 June) and end (3–23 July) of the second half of the summer term when primary schools were partially reopened and after full reopening of all schools in September 2020, at the end of the autumn term (23 November–18 December). Samples for extended humoral and cellular analysis were taken in round 3. Additional samples from children found to be seropositive in round 1 were taken from 21 June to 24 July 2021. No statistical methods were used to predetermine sample sizes. Researchers were blinded to the serostatus of donors before ELISpot and serological assessment.

For each known SARS-CoV-2 seropositive individual, an age-matched (nearest age in years for students, nearest ten years for teachers) and sex-matched participant also underwent blood sampling. In total 154 adults and 91 children had sufficient blood sample for serology and cellular responses (Table 1). Convalescent plasma samples were also available from 35 children aged 10–13 years with PCR-confirmed SARS-CoV-2 infection, taken a median of 6 months (range 2–12 months) after the PCR result, from the Born in Bradford study^36^.

**Table 1:** Demographics and serostatus of study participants

	*n*	Age in years, median (range)	Sex	Infection >6 months^a^
**Adults**				
**All**	154	40 (20–71)	19% Male 81% Female	–
**Seropositive**	91	39 (20–71)	18% Male 82% Female	81
**Seronegative**	63	43 (26–65)	21% Male 79% Female	–
**Children**				
**All**	91	7 (3–11)	52% Male 48% Female	–
**Seropositive**	43	8 (5–11)	67% Male 33% Female	35
**Seronegative**	48	7 (3–11)	53% Male 47% Female	–

^a^Seropositive at first assessment (June 2020).

Prepandemic plasma and PBMCs were obtained from healthy children as part of an ethically approved study (TrICICL) (South of Birmingham Research Ethics Committee 17/WM/0453, Integrated Research Application System 233593). Ethical review for the current study was provided by the PHE Research Ethics and Governance Group (PHE R&D REGG ref. no. NR0209). Written informed consent was obtained from all adult participants and from parents or guardians.

### PBMC and plasma preparation

Blood tubes were spun at 300*g* for 10 min before removal of plasma, which was then spun again at 800*g* for 10 min and stored at −80 °C. The remaining blood was diluted 1:1 with Roswell Park Memorial Institute (RPMI) medium and PBMCs were isolated on a SepMate (STEMCELL Technologies) density centrifugation tube, washed with RPMI and rested in RPMI + 10% FCS overnight at 37 °C.

### MSD serology assay

Quantitative immunoglobulin G (IgG) antibody titers were measured against trimeric spike protein, nucleocapsid and other coronavirus using the MSD V-PLEX COVID-19 Coronavirus Panel 2 (N05368-A1); Coronavirus Panel 7 (N05428A-1) responses to other respiratory viruses were measured using the MSD V-PLEX COVID-19 Respiratory Panel 1 (N05358-A1). Multiplex MSD assays were performed according to the manufacturer’s instructions. Briefly 96-well plates were blocked. After washing, samples diluted 1:5,000 in diluent, as well as reference standard and internal controls, were added to the wells. After incubation plates were washed and detection antibody added. Plates were washed and were immediately read using a MESO TM QuickPlex SQ 120 system. Data were generated by Methodological Mind software v.4.0 and analyzed with the MSD Discovery Workbench v.4.0 software. Assay cutoffs with regard to prepandemic plasma samples from healthy donors are shown in Extended Data 1. Cutoffs used were 350 a.u. ml^−1^ for spike, 600 a.u. ml^−1^ for RBD and nucleocapsid and 15 a.u. ml^−1^ for NTD.

### Total IgG/A/M anti-spike SARS-CoV-2 ELISA

A total IgG/A/M anti-SARS-CoV-2 spike ELISA kit^37^ was purchased from Binding Site. ELISA was performed according to the manufacturer’s instructions. Optical density was compared to a known calibrator and expressed as a ratio to the calibrator. Samples with a ratio >1.0 were considered seropositive.

### Cross-reactive antibody blocking

Plasma samples were prediluted 1:10 with PBS then preabsorbed by adding an equal volume of either recombinant spike S1 domain (catalog no. 10569-CV-100; R&D Systems) or spike S2 domain (catalog no. 10594-CV-100; R&D Systems) at a concentration of 500 μg ml^−1^ in PBS or PBS alone (mock). Samples were incubated at 37 °C for 30 min. Samples were then diluted to a final dilution of 1:5,000 in MSD diluent and run on an MSD V-PLEX COVID-19 Respiratory Panel 2 plate in duplicate.

### RBD-ACE2 competitive binding assay

The concentration of antibodies that inhibited the interaction between RBD and ACE2 was measured using a SARS-CoV-2 neutralization assay (BioLegend) according to the manufacturer’s instructions. Briefly, plasma or positive control antibody were preincubated with biotinylated-Fc-chimera-S1-RBD protein before adding bead-bound ACE2. Binding of RBD to ACE2 was then measured by adding streptavidin-phycoerythrin. Samples were run on a BD LSR II flow cytometer and analyzed using the LEGENDplex v.8.0 software (BioLegend). Results were related to a known RBD neutralizing antibody standard and displayed as ng ml^−1^.

### Live virus and pseudotype-based neutralization assays

The clinical isolates used in the study were provided by PHE and Imperial College London.

A549-ACE2-TMPRSS2 cells^38^ were seeded at a cell density of 1 × 10^4^ per well in 96-well plates 24 h before inoculation. Serum was titrated starting at a 1:100 dilution. The specified virus was then incubated at a multiplicity of infection of 0.01 with the serum for 1 h before infection. All wells were performed in triplicate; 72 h later infection plates were fixed with 8% formaldehyde and stained with Coomassie brilliant blue dye for 30 min. Plates were washed and dried overnight before quantification using a Celigo Imaging Cytometer (Nexcelom Bioscience) to measure staining intensity. Percentage cell survival was assessed by comparing the intensity of the staining to uninfected wells.

HEK293, HEK293T and 293-ACE2 cells were maintained in DMEM supplemented with 10% FCS, 200 mM of L-glutamine, 100 µg ml^−1^ streptomycin and 100 IU ml^−1^ penicillin. HEK293T cells were transfected with the appropriate SARS-CoV-2 S gene expression vector in conjunction with lentiviral vectors p8.91 and pCSFLW using polyethylenimine (Polysciences). Human immunodeficiency virus (HIV) (SARS-CoV-2) pseudotype-containing supernatants were collected 48 h posttransfection, aliquoted and frozen at −80 °C before use. The SARS-CoV-2 spike glycoprotein expression constructs for Wuhan-Hu-1, B.1.351 (South Africa) and B.1.617.2 have been described elsewhere^39^. Constructs bore the following mutations relative to the Wuhan-Hu-1 sequence (GenBank ID: MN908947): B.1.351—D80A, D215G, L241-243del, K417N, E484K, N501Y, D614G and A701V; B.1.617.2—T19R, G142D, E156del, F157del, R158G, L452R, T478K, D614G, P681R and D950N. 293-ACE2 target cells were maintained in complete DMEM supplemented with 2 µg ml^−1^ puromycin.

Neutralizing activity in each sample was measured by a serial dilution approach. Each sample was serially diluted in triplicate from 1:50 to 1:36,450 in complete DMEM before incubation with approximately 1 × 10^6^ counts per s per well of HIV (SARS-CoV-2) pseudotypes, incubated for 1 h and plated onto 239-ACE2 target cells. After 48–72 h, luciferase activity was quantified by adding Steadylite Plus chemiluminescence substrate and analysis on a PerkinElmer EnSight multimode plate reader. Antibody titer was then estimated by interpolating the point at which infectivity had been reduced to 90% of the value for the no serum control samples.

### Interferon-γ ELISpot

A pepmix pool containing 15-mer peptides overlapping by 10 amino acids from either the SARS-CoV-2 spike S1 or S2 domains and a combined pool of nucleoprotein and membrane and envelope were purchased from Alta Bioscience. Overlapping pepmixes from influenza A matrix protein 1, California/08/2009 (H1N1) (protein ID: C3W5Z8) and A/Aichi/2/1968 H3N2 (protein ID: Q67157) were purchased from JPT Peptide Technologies and combined as a relevant control.

T cell responses were measured using an interferon-γ (IFN-γ) ELISpot Pro Kit (Mabtech) as described previously^33^. Briefly, fresh PBMCs were rested overnight before assay and 0.25–0.3 × 10^6^ PBMCs were added in duplicate per well containing either pepmix, anti-CD3 (positive) or dimethyl sulfoxide (DMSO) (negative) control. Samples were incubated for 16–18 h. The supernatant was collected and stored at −80 °C. Plates were developed according to the manufacturer’s instructions and read using an AID plate reader (AID). Cutoff values were determined previously^33^.

### Cross-reactive T cell assay

A total of 1.3 × 10^6^ PBMCs were peptide-pulsed with SARS-CoV-2 S2 pepmix pool (JPT Peptide Technologies) at a concentration of 1 μg ml^−1^ per peptide or an equal volume of DMSO. Cells were then plated into a 48-well plate and cultured in RPMI + 10% FCS + penicillin/streptomycin with the addition of 20 U ml^−1^ of IL-2 for 9 days, with frequent media changes. IL-2 was removed 24 h before the assay. Cells were washed and divided across four wells of an ELISpot plate and then restimulated with either SARS-CoV-2 S2 pepmix or S2 pepmixes from either the Beta (OC43 and HKU-1) or Alpha (NL63 and 229E) hCoVs. The results were read as for ELISpot and presented as expansion compared to the DMSO controls.

### Cytokine measurement

Supernatants from donors with a detectable response in overnight ELISpot cultures were assessed using a LEGENDplex Th-profile 12-plex Kit (BioLegend) according to the manufacturer’s instructions. Data were analyzed with the LEGENDplex v.8.0 software.

### Intracellular cytokine staining

Cryopreserved PBMCs were thawed and rested overnight. Cells were then stimulated with a combined spike S1 and S2 peptide pool, at a final concentration of 1 μg ml^−1^ per peptide or DMSO (mock). After 1 h, eBioscience protein transport inhibitor cocktail (Thermo Fisher Scientific) was added and cells were incubated for a further 5 h. eBioscience cell stimulation cocktail (Thermo Fisher Scientific) was used as a positive control. After stimulation, cells were washed (PBS + 0.1% BSA) and surface-stained at 4 °C for 30 min. Cells were then washed and fixed in 2% paraformaldehyde. After washing, brilliant staining buffer (BD Bioscience) and a final concentration of 0.4% saponin were added. Cells were stained intracellularly at room temperature for 30 min. Cells were then washed and run on a BD FACSymphony A3 flow cytometer (BD Biosciences). Antibody details are provided in Supplementary Table 4.

### Data visualization and statistics

Statistical tests, including normality tests, were performed as indicated using Prism 9 (GraphPad Software). Only significant results (*P* < 0.05) are displayed.

### Reporting Summary

Further information on research design is available in the Nature Research Reporting Summary linked to this article.

## Online content

Any methods, additional references, Nature Research reporting summaries, source data, extended data, supplementary information, acknowledgements, peer review information; details of author contributions and competing interests; and statements of data and code availability are available at 10.1038/s41590-021-01089-8.

## Supplementary information

Supplementary Information Supplementary Tables 1–4.

Reporting Summary

Peer Review Information

## Data Availability

All data in this study are available within the article and its supplementary files and from the corresponding author upon reasonable request. Source data are provided with this paper.

## Source data

Source Data Fig. 1 Statistical source data for Fig. 1.
Source Data Fig. 2 Statistical source data for Fig. 2.
Source Data Fig. 3 Statistical source data for Fig. 3.
Source Data Fig. 4 Statistical source data for Fig. 4.
Source Data Fig. 5 Statistical source data for Fig. 5.
Source Data Fig. 6 Statistical source data for Fig. 6.
Source Data Extended Data Fig. 1 Statistical Source Data for Extended Data Fig. 1.
Source Data Extended Data Fig. 2 Statistical Source Data for Extended Data Fig. 2.
Source Data Extended Data Fig. 3 Statistical Source Data for Extended Data Fig. 3.

## References

[1] Booth A (2021). Population risk factors for severe disease and mortality in COVID-19: a global systematic review and meta-analysis. PLoS ONE.

[2] Viner RM (2021). Susceptibility to SARS-CoV-2 infection among children and adolescents compared with adults: a systematic review and meta-analysis. JAMA Pediatr..

[3] Weisberg SP (2021). Distinct antibody responses to SARS-CoV-2 in children and adults across the COVID-19 clinical spectrum. Nat. Immunol..

[4] Pierce CA (2020). Immune responses to SARS-CoV-2 infection in hospitalized pediatric and adult patients. Sci. Transl. Med..

[5] Tosif S (2020). Immune responses to SARS-CoV-2 in three children of parents with symptomatic COVID-19. Nat. Commun..

[6] Cohen CA (2021). SARS-CoV-2 specific T cell responses are lower in children and increase with age and time after infection. Nat. Commun..

[7] Flood J (2021). Paediatric multisystem inflammatory syndrome temporally associated with SARS-CoV-2 (PIMS-TS): prospective, national surveillance, United Kingdom and Ireland, 2020. Lancet Reg. Health Eur..

[8] Consiglio CR (2020). The immunology of multisystem inflammatory syndrome in children with COVID-19. Cell.

[9] Hoang A (2020). COVID-19 in 7780 pediatric patients: a systematic review. EClinicalMedicine.

[10] Gruber CN (2020). Mapping systemic inflammation and antibody responses in multisystem inflammatory syndrome in children (MIS-C). Cell.

[11] *Protein BLAST* (National Center for Biotechnology Information, 2021); https://blast.ncbi.nlm.nih.gov/Blast.cgi?PROGRAM=blastp&PAGE_TYPE=BlastSearch&LINK_LOC=blasthome

[12] Dijkman R (2008). Human coronavirus NL63 and 229E seroconversion in children. J. Clin. Microbiol..

[13] Huang AT (2020). A systematic review of antibody mediated immunity to coronaviruses: kinetics, correlates of protection, and association with severity. Nat. Commun..

[14] Dijkman R (2012). The dominance of human coronavirus OC43 and NL63 infections in infants. J. Clin. Virol..

[15] Ng KW (2020). Preexisting and de novo humoral immunity to SARS-CoV-2 in humans. Science.

[16] Ladhani SN (2021). SARS-CoV-2 infection and transmission in primary schools in England in June–December, 2020 (sKIDs): an active, prospective surveillance study. Lancet Child Adolesc. Health.

[17] Sabino EC (2021). Resurgence of COVID-19 in Manaus, Brazil, despite high seroprevalence. Lancet.

[18] Bates TA (2021). Neutralization of SARS-CoV-2 variants by convalescent and BNT162b2 vaccinated serum. Nat. Commun..

[19] Ju B (2020). Human neutralizing antibodies elicited by SARS-CoV-2 infection. Nature.

[20] Madera S (2021). Nasopharyngeal SARS-CoV-2 viral loads in young children do not differ significantly from those in older children and adults. Sci. Rep..

[21] Neeland MR (2021). Innate cell profiles during the acute and convalescent phase of SARS-CoV-2 infection in children. Nat. Commun..

[22] Anderson EM (2021). Seasonal human coronavirus antibodies are boosted upon SARS-CoV-2 infection but not associated with protection. Cell.

[23] Westerhuis, B. M. et al. Severe COVID-19 patients display a back boost of seasonal coronavirus-specific antibodies. Preprint at 10.1101/2020.10.10.20210070 (2020).

[24] Chan KH (2005). Serological responses in patients with severe acute respiratory syndrome coronavirus infection and cross-reactivity with human coronaviruses 229E, OC43, and NL63. Clin. Diagn. Lab. Immunol..

[25] Khan T (2021). Distinct antibody repertoires against endemic human coronaviruses in children and adults. JCI Insight.

[26] Shrock E (2020). Viral epitope profiling of COVID-19 patients reveals cross-reactivity and correlates of severity. Science.

[27] Meade P (2020). Influenza virus infection induces a narrow antibody response in children but a broad recall response in adults. mBio.

[28] Bartsch YC (2021). Humoral signatures of protective and pathological SARS-CoV-2 infection in children. Nat. Med..

[29] Mateus J (2020). Selective and cross-reactive SARS-CoV-2 T cell epitopes in unexposed humans. Science.

[30] Nelde A (2021). SARS-CoV-2-derived peptides define heterologous and COVID-19-induced T cell recognition. Nat. Immunol..

[31] Saletti G (2020). Older adults lack SARS CoV-2 cross-reactive T lymphocytes directed to human coronaviruses OC43 and NL63. Sci. Rep..

[32] Reynolds CJ (2020). Discordant neutralizing antibody and T cell responses in asymptomatic and mild SARS-CoV-2 infection. Sci. Immunol..

[33] Zuo J (2021). Robust SARS-CoV-2-specific T-cell immunity is maintained at 6 months following primary infection. Nat. Immunol..

[34] Dan JM (2021). Immunological memory to SARS-CoV-2 assessed for up to 8 months after infection. Science.

[35] Shaman J, Galanti M (2020). Will SARS-CoV-2 become endemic?. Science.

[36] Bird PK (2019). Growing up in Bradford: protocol for the age 7–11 follow up of the Born in Bradford birth cohort. BMC Public Health.

[37] Cook AM (2021). Validation of a combined ELISA to detect IgG, IgA and IgM antibody responses to SARS-CoV-2 in mild or moderate non-hospitalised patients. J. Immunol. Methods.

[38] Rihn SJ (2021). A plasmid DNA-launched SARS-CoV-2 reverse genetics system and coronavirus toolkit for COVID-19 research. PLoS Biol..

[39] Davis, C. et al. Reduced neutralisation of the Delta (B.1.617.2) SARS-CoV-2 variant of concern following vaccination. Preprint at 10.1101/2021.06.23.21259327 (2021). 10.1371/journal.ppat.1010022 PMC8639073 34855916

