# Supplementary material for: Children develop robust and sustained cross-reactive spike-specific immune responses to SARS-CoV-2 infection
Source: Nat Immunol. 2021 Dec 22;23(1):40–9. doi: 10.1038/s41590-021-01089-8 (PMC8709786; doi:10.1038/s41590-021-01089-8)
Supplement: Supplementary file 1 — Supplementary Tables 1–4. [file 41590_2021_1089_MOESM1_ESM.pdf]

nature portfolio

https://doi.org/10.1038/s41590-021-01089-8

Supplementary information

Children develop robust and sustained cross-reactive spike-specific immune responses to SARS-CoV-2 infection

In the format provided by the authors and unedited

		Children		Adult		Pre-COVID	
		Seropositive*	Seronegative*	Seropositive*	Seronegative*	Children	Adult
MSD serology[1]	SARS-CoV-2 Spike	11589±4.097 (43/43)	57.43±2.178 (0/48)	8593±4.173 (91/91)	61.14 ±2.047 (0/63)	44.34±3.719 (0/9)	13.22±4.893 (0/50)
	SARS-CoV-2 Nucleoprotein	5914±3.735 (40/43, 93%)	160.4±3.625 (7/48, 15%)	5321±5.116 (85/90, 94%)	216.6±5.038 (17/63, 27%)	68.71±2.764 (0/9)	76.238±6.238 (6/50, 12%)
	SARS-CoV-2 RBD	4527±3.673 (40/43, 93%)	70.80±2.435 (1/48, 2%)	2845±4.755 (79/91, 87%)	106.0±2.604 (2/63, 5%)	49./68±3.496 (0/9)	6.085±4.865 (0/50)
	SARS-CoV-2 NTD	234.9±3.436 (41/43, 95%)	2.119±2.630 (1/47, 2%)	106.9±6.290 (79/91, 87%)	2.271±3.265 (2/60, 3%)	1.757±4.179 (1/9, 11%)	1.196±1.99 (0/11)
	OC43	36938±2.780 (43)	19296±2.531 (48)	38644±2.151 (91)	30526±1.974 (63)	n.a.	
	HKU-1	8014±2.346 (43)	3544±3.181 (48)	12810±2.402 (91)	9090±2.698 (63)		
	NL63	2861±2.482 (43)	1899±2.599 (48)	3112±1.914 (91)	2643±2.304 (63)		
	229e	11327±5.498 (43)	4845±6.982 (48)	22822±2.273 (91)	17032±2.951 (63)		
ELISpot[2]	Spike (S1+S2)	532.9±591.0 (32/37, 86%)	212.7±242.6 (12/20, 60%)	195.0±280.7 (45/64, 69%)	111.7±244.1 (10/29, 34%)		
	N/M	33.81±43.27 (16/37, 44%)	4.172±8.574 (2/20, 10%)	75.91±147.9 (40/64, 63%)	19.59±83.46 (2/29, 7%)		
	Flu	110.0±160.9 (37)	159.2±243.0 (18)	147.6±369.9 (52)	164.4±275.1 (24)		
**+6mth Infection[3]**							
MSD serology	SARS-CoV-2 Spike	11450±4.428 (34/35, 97%)	n.d.	6102±7.131 (73/81, 90%)	n.a.	n.a.	
	SARS-CoV-2 Nucleoprotein	6086±2.26 (35/35, 100%)		4611±5.45 (75/81, 93%)			
	SARS-CoV-2 RBD	4366±(3.624) (33/35, 94%)		2482±5.029 (69/81, 85%)			
ELISpot	Spike (S1+S2)	530.8±600.7 (23/27, 85%)		176.1±228.9 (37/52, 71%)			
	N/M	35.41±42.51 (12/27, 44%)		86.44±161.7 (35/52, 67%)			
	Neutralising antibody (ng/ml)	278.9±1.485 (12)		247.4±1.608 (15)		n.a.	n.d. (10)

Supplementary Table 1. Immunological Characteristics of Seropositive and Seronegative Children and Adults. MSD serology data is presented as Geometric mean ±SD (AU/ml), ELISpot data is presented as mean±SD (sfc/10^6 PBMC). *Seropositive/negative defined by 1. MSD-anti-spike titre, 2. MSD-anti-Spike or prior PHE seropositive or total IgG/A/M anti-spike ELISA. 3. PHE sKIDS prior positive (June-July 2020). N.d. – not detected.

		ELISpot (sfc/10^6 PBMC)			MSD Serology – AU/ml										
**Adult /Child**	**Age**	**Spike**	**N/M**	**Flu**	**COV-2-Spike**	**COV-2-RBD**	**COV-2 NTD**	**COV2-Nucleocapsid**	**OC43 Spike**	**HKU-1 Spike**	**NL63 Spike**	**229e Spike**	**sKIDS round 1**	**sKIDS round 2**	**IgG,A,M ELISA**
Child	9	**110**	4	172	5	285	2	50	28273	***12195***	2925	***87935***	Negative	Negative	Negative
Child	5	**243**	0	0	12	10	1	546	20067	1650	2012	432	Negative	Negative	Negative
Child	7	**418**	0	766	39	14	0	29	***31263***	***16729***	3684	***18035***	Negative		Negative
Child	11	**70**	0	796	73	225	2	**603**	7360	5213	1263	***60299***	Negative	Negative	Negative
Child	10	**142**	0	230	40	25	0	59	23156	5741	4402	8572	Negative	Negative	Negative
Child	11	**274**	0	276	127	97	4	56	***53420***	3992	***7148***	***14452***			Negative
Child	5	**330**	6	54	134	171	3	241	12221	1481	4213	***37470***	Negative	Negative	Negative
Child	8	**168**	0	4	220	47	2	43	***29509***	5580	675	***12144***	Negative	Negative	Negative
Child	5	**742**	2	36	25	37	1	93	6215	2197	1425	2828	Negative	Negative	Negative
Child	5	**776**	**32**	16	42	40	2	51	2637	268	305	153	Negative	Negative	Negative
Child	5	**484**	8	136	20	58	1	42	2610	2239	1072	6684	Negative		Negative
Child	7	**387**	**24**	104	109	36	1	**1465**	9255	***8513***	4582	5311	Negative	Negative	Negative
Staff	49	**148**	0	8	56	323	5	51	43504	11602	2468	28990	Negative		Negative
Staff	38	**62**	0	430	69	114	2	46	***106800***	***26036***	***7039***	***37074***	Negative	Negative	Negative
Staff	43	**496**	2	1246	64	132	0	299	30002	9771	2877	19008	Negative	Negative	Negative
Staff		**37**	1	19	84	75	3	105	43170	3686	4180	12688	Negative	Negative	Negative
Staff	47	**160**	0	392	225	136	7	**804**	23610	5171	826	29936	Negative	Negative	Negative
Staff	38	**1124**	16	374									Negative	Negative	Negative
Staff	65	**524**	2	320	56	39	1	150	***62856***	***25802***	2758	**33779**	Negative	Negative	Negative
Staff	31	**84**	4		65	55	2	278	***63770***	9017	3795	24533	Negative		Negative
Staff	52	6	**32**	96	62	64	12	60	30596	6158	***5140***	2549	Negative	Negative	Negative
Staff	31	8	**452**	64	33	395	8	22	25250	1613	692	***46524***	Negative	Negative	Negative
Staff	53	**428**	2		46	151	1	49	29970	***18828***	***4699***	***35577***	Negative		Negative

Supplementary Table 2. Immune characteristics of ELISpot Responding Seronegative donors.

Bold values indicate values above cut-off. ***Bold-italicised*** indicates values +50% higher than geometric mean**.**

		Wuhan		B.1.1.7		B1.351		P.1	
		Children	Adults	Children	Adults	Children	Adults	Children	Adults
SARS-CoV-2 Spike	Geo. Mean Titre ±SD (AU/ml)	13138 ±2.1	6701 ±2.1	11067 ±2.0	6351 ±2.1	6862 ±2.1	3766 ±2.3	8477 ±2.1	4115 ±2.0
	Normalised Titre* Median (95%CI)	-	-	82% (75-89)	94% (82-109)	49% (46-59)	53% (46-73)	63% (59-66)	64% (53-74)
SARS-CoV-2 RBD	Geo. Mean Titre ±SD (AU/ml)	4198 ±2.4	1954 ±2.4	2938 ±2.4	1370 ±2.6	1299 ±2.3	473.3 ±2.4	2018 ±2.5	691 ±2.5
	Normalised Titre* Median (95%CI)	-	-	71% (68-72)	72% (66-74)	31% (29-34)	27% (21-30)	47% (43-52)	39% (31-44)
	Seropositive donors† (%)	19/19 (100%)	17/18 (94%)	19/19 (100%)	16/18 (89%)	16/19 (84%)	5/18 (28%)	16/19 (84%)	8/18 (44%)

Supplementary Table 3. Antibody reactivity against SARS-CoV-2 spike and RBD from variants of concern in 6+-month convalescent children and adults. *Relative percentage of binding to Wuhan viral protein. †seropositivity defined based on a cut-off of 600 AU/ml.

Antigen	Clone	Fluorophore	Supplier	Code	Dilution
eBioscience Fixable Viability Dye	-	eFluor 780	ThermoFisher Scientific	65-0865-14	1/10000
CD14	HCD14	APC-Cy7	Biolegend	325620	1/100
CD19	HIB19	APC-Cy7	Biolegend	302218	1/100
CD3	UCHT1	Fitc	Biolegend	300406	1/50
CD4	RPAT4	Per-CP-Cy5.5	Biolegend	300529	1/50
CD8	SK1	BV510	Biolegend	344732	1/50
IL-2	MQ117H12	Pe-Cy7	Biolegend	500326	1/50
IL-4	MP4 25D2	APC	Biolegend	500813	1/25
IL-6	MQ2 13A5	PE-Dazzle 594	Biolegend	501122	1/20
IL-10	Jes3-9D7	PE	Biolegend	501404	1/20
IL-17A	BL168	BV421	Biolegend	512322	1/20
IFN-γ	4S.B3	AF700	Biolegend	502520	1/100
TNF	Ab11	BV711	Biolegend	502940	1/20

Supplementary Table 4. Flow cytometry antibodies
