# Supplementary material for: Children develop robust and sustained cross-reactive spike-specific immune responses to SARS-CoV-2 infection
Source: Nat Immunol. 2021 Dec 22;23(1):40–9. doi: 10.1038/s41590-021-01089-8 (PMC8709786; doi:10.1038/s41590-021-01089-8)
Supplement: Supplementary file 2 — Reporting Summary [file 41590_2021_1089_MOESM2_ESM.pdf]

nature research

Corresponding author(s): Prof. Paul Moss

Last updated by author(s): Oct 13, 2021

Reporting Summary

Nature Research wishes to improve the reproducibility of the work that we publish. This form provides structure for consistency and transparency in reporting. For further information on Nature Research policies, see our Editorial Policies and the Editorial Policy Checklist.

Statistics

For all statistical analyses, confirm that the following items are present in the figure legend, table legend, main text, or Methods section.

n/a	Confirmed	
☐	☒	The exact sample size (*n*) for each experimental group/condition, given as a discrete number and unit of measurement
☐	☒	A statement on whether measurements were taken from distinct samples or whether the same sample was measured repeatedly
☐	☒	The statistical test(s) used AND whether they are one- or two-sided<br>*Only common tests should be described solely by name; describe more complex techniques in the Methods section.*
☐	☒	A description of all covariates tested
☐	☒	A description of any assumptions or corrections, such as tests of normality and adjustment for multiple comparisons
☐	☒	A full description of the statistical parameters including central tendency (e.g. means) or other basic estimates (e.g. regression coefficient) AND variation (e.g. standard deviation) or associated estimates of uncertainty (e.g. confidence intervals)
☐	☒	For null hypothesis testing, the test statistic (e.g. *F*, *t*, *r*) with confidence intervals, effect sizes, degrees of freedom and *P* value noted<br>*Give P values as exact values whenever suitable.*
☒	☐	For Bayesian analysis, information on the choice of priors and Markov chain Monte Carlo settings
☒	☐	For hierarchical and complex designs, identification of the appropriate level for tests and full reporting of outcomes
☒	☐	Estimates of effect sizes (e.g. Cohen's *d*, Pearson's *r*), indicating how they were calculated

Our web collection on statistics for biologists contains articles on many of the points above.

Software and code

Policy information about availability of computer code

Data collection	Methodological Mind software; Meso Scale Diagnostics Workbench version 4, BD FACSDiva Version 9.0. AID ELISpot software version 8.
Data analysis	Meso Scale Diagnostics Workbench version 4, Flow Jo Version 10.6.1, Graphpad Prism Version 9, Legendplex Software Version 8.

For manuscripts utilizing custom algorithms or software that are central to the research but not yet described in published literature, software must be made available to editors and reviewers. We strongly encourage code deposition in a community repository (e.g. GitHub). See the Nature Research guidelines for submitting code & software for further information.

Data

Policy information about availability of data

All manuscripts must include a data availability statement. This statement should provide the following information, where applicable:

- Accession codes, unique identifiers, or web links for publicly available datasets
- A list of figures that have associated raw data
- A description of any restrictions on data availability

The data that support the findings of this study are available from the supporting data source files.

Field-specific reporting

Please select the one below that is the best fit for your research. If you are not sure, read the appropriate sections before making your selection.

☒ Life sciences ☐ Behavioural & social sciences ☐ Ecological, evolutionary & environmental sciences

For a reference copy of the document with all sections, see nature.com/documents/nr-reporting-summary-flat.pdf

Life sciences study design

All studies must disclose on these points even when the disclosure is negative.

Sample size	All Adult and Children found to be SARS-CoV-2 sero-positive in the prior sKIDS study were invited to participate further for this study, all donors willing to participate were included. Matched donors of a similar age who were previously sero-negative were also asked to participate.
Data exclusions	Donors were excluded from ELISpot for the following reasons, a) insufficient cell number, b) failure of positive control wells, c) high background in the negative control wells. Data were excluded from the cross-reactive spike blocking assay if a reduction of SARS-CoV-2 Spike antibody level was not evident. Data was excluded from MSD analysis if multiple spots failed to give signal to antigens of which response would be expected i.e. hCoV.
Replication	Due to the limitations of blood volume it was not possible to repeat cellular assays. Serological assays were assessed by multiple methods as stated.
Randomization	All donor were treated equally. Researchers were blinded to the serostatus of donors until initial MSD and ELISpot data acquisition had been completed.
Blinding	Researchers were blinded to the serostatus of donors until initial MSD and ELISpot data acquisition had been completed.

Reporting for specific materials, systems and methods

We require information from authors about some types of materials, experimental systems and methods used in many studies. Here, indicate whether each material, system or method listed is relevant to your study. If you are not sure if a list item applies to your research, read the appropriate section before selecting a response.

Materials & experimental systems

n/a	Involved in the study	
☐	☒	Antibodies
☐	☒	Eukaryotic cell lines
☒	☐	Palaeontology and archaeology
☒	☐	Animals and other organisms
☐	☒	Human research participants
☒	☐	Clinical data
☒	☐	Dual use research of concern

Methods

n/a	Involved in the study	
☒	☐	ChIP-seq
☐	☒	Flow cytometry
☒	☐	MRI-based neuroimaging

Antibodies

Antibodies used

CD14 clone HCD14 APC-Cy7 Biolegend cat 325620 lot B282904
CD19 clone HIB19 APC-Cy7 Biolegend cat 302218 lot B279663
CD3 clone UCHT1 Fitc Biolegend cat 300406 lot B279208
CD4 clone RPAT4 Per-CP-Cy5.5 Biolegend cat 300529 lot B277600
CD8 clone SK1 BV510 Biolegend cat 344732 lot B270694
IL-2 clone MQ117H12 Pe-Cy7 Biolegend cat 500326 lot B314085
IL-4 clone MP4 25D2 APC Biolegend cat 500813 lot B262980
IL-6 clone MQ2 13A5 PE-Dazzle 594 Biolegend cat 501122 lot B284858
IL-10 clone Jes3-9D7 PE Biolegend cat 501404 lot B285627
IL-17A clone BL168 BV421 Biolegend cat 512322 lot B317903
IFNγ clone 4S.B3 AF700 Biolegend cat 502520 lot B302043
TNF clone Ab11 BV711 Biolegend cat 502940 B326604

Validation

These antibodies and intracellular cytokine staining are standardly used. Citing references and application references, alongside example staining are provided at www.Biolegend.com. Cell lineage markers (i.e. CD14, CD19, CD3, CD4, CD8) were titrated alone or in combination using health donor PBMC, to obtain concentrations which clearly identified populations of interest, and ensure staining of the negative population was not present. Intracellular antibodies for this panel were titrated and tested on healthy donor

PBMC using PMA/Ionomycin and widely recognized peptide antigen (CEFX), cells were stimulated as described in methods, PMA stimulates cytokine production by T cells irrespective of antigen recognition, as such PBMC produce the cytokines listed (examples of PMA induced cytokine production for each of the antibodies can be found on the product page at www.biolegend.com). Following surface staining for the linage markers previously optimised. Titration of the various antibodies was then preformed to determine the optimal dilution to provided resolution of cytokine producing cells, unstimulated cells were used to ensure specificity and absence of staining in the negative population. Examples of staining and gating are provided in Extended Data Figure 2. A positive control using PMA/Ionomycin was included in all experiments.

Eukaryotic cell lines

Policy information about cell lines

Cell line source(s)	HEK-293 were obtained circa 2000 from Yasu Takeuchi, University College London.
Authentication	HEK-293 have not been formally identified, but have been in the possession of Brian Willet and used by Brian Willet since 2000.
Mycoplasma contamination	All cell lines were routinely tested for mycoplasma and are negative.
Commonly misidentified lines (See ICLAC register)	Not on the registry.

Human research participants

Policy information about studies involving human research participants

Population characteristics	Previously sero-positive donors (Children aged 3-11 and adults aged 20-71) were age and gender matched as closely as possible, (maximally within 1yr for children, 10 yrs for adults). Age and gender for the sero-postive and negative cohorts are provided in table 1. Full characteristics of the sKIDS cohort are provided in the sKIDS study protocol and publication (https://www.gov.uk/guidance/covid-19-paediatric-surveillance, Ladhani, S.N. et al. SARS-CoV-2 infection and transmission in primary schools in England in June-December, 2020 (sKIDs): an active, prospective surveillance study. The Lancet. Child & adolescent health (2021)).
Recruitment	Adult and Children found to be SARS-CoV-2 sero-positive in the prior sKIDS study were invited to participate further for this study, only Sero-positive donors reporting mild or asymptomatic infection were included in the collection. All donors willing to participate were included. Matched donors of a similar age who were previously sero-negative were also asked to participate. These donors are the most relevant control group as such no bias should be evident.
Ethics oversight	Written informed consent was obtained from all donors, either directly from adults (aged >18 yrs old) or from legally authorized representatives of minor participants (age below <18 yrs old). Ethical approval was obtained from PHE Research Ethics Governance Group (reference NR0209; May 16, 2020), South of Birmingham Research Ethics Committee (REC: 17/WM/0453, IRAS: 233593), Bradford Research Ethics Committee (Ref 07/H1302/112).

Note that full information on the approval of the study protocol must also be provided in the manuscript.

Flow Cytometry

Plots

Confirm that:

☒ The axis labels state the marker and fluorochrome used (e.g. CD4-FITC).

☒ The axis scales are clearly visible. Include numbers along axes only for bottom left plot of group (a 'group' is an analysis of identical markers).

☒ All plots are contour plots with outliers or pseudocolor plots.

☒ A numerical value for number of cells or percentage (with statistics) is provided.

Methodology

Sample preparation	PBMC was prepared as described, excess cells were cryopreserved in 10%DMSO/90% FBS, and stored in the vapor phase of liquid nitrogen. Cells were thawed by warming at 37oC and washed in warmed media. Cells were rested overnight prior to experiment.
Instrument	28 color BD FACS Symphony A3 Flow cytometer.
Software	BD FACS Diva Version 9.
Cell population abundance	Cell sorting was not preformed, analysis by flow cytometery only. Observed cytokine producing cells in response to SARS-CoV-2 Spike peptide pool ranged from 0.677% of parent cell population (CD8 T cells) to >0.001%, dependent upon cytokine produced, response was determined against abundance in a DMSO stimulated negative control, as shown in Extended Data Figure 2.

Gating strategy

Lymphocytes were gated by FSC/SSC, followed by doublet exclusion by FSC-A/FSC-H. Live CD3+ CD14- CD19- cells were then gated, and CD4+CD8- or CD4-CD8+ T cells were finally gated.

☒ Tick this box to confirm that a figure exemplifying the gating strategy is provided in the Supplementary Information.
