# Supplementary material for: Children develop robust and sustained cross-reactive spike-specific immune responses to SARS-CoV-2 infection
Source: Nat Immunol. 2021 Dec 22;23(1):40–9. doi: 10.1038/s41590-021-01089-8 (PMC8709786; doi:10.1038/s41590-021-01089-8)
Supplement: Supplementary file 3 — Peer Review Information [file 41590_2021_1089_MOESM3_ESM.pdf]

natureresearch

# Peer Review Information

**Journal:** Nature Immunology
**Manuscript Title:** Children develop strong and sustained cross-reactive immune responses against Spike protein following SARS-CoV-2 infection, with enhanced recognition of variants of concern
**Corresponding author name(s):** Paul Moss

## Reviewer Comments & Decisions:

**Decision Letter, initial version:**

**Subject:** Decision on Nature Immunology submission NI-A32053
**Message:** 14th Jun 2021

Dear Professor Moss,

Your Article, "Children develop strong and sustained cross-reactive immune responses against Spike protein following SARS-CoV-2 infection, with enhanced recognition of variants of concern" has now been seen by 3 referees as you are aware. Their comments are below. Many thanks for supplying an author repsonse to these comments. We are very interested in the possibility of publishing your study in Nature Immunology if you are able to revise it in line with your suggestions.

We therefore invite you to revise your manuscript taking into account all reviewer and editor comments. Please highlight all changes in the manuscript text file in Microsoft Word format.

We are committed to providing a fair and constructive peer-review process. Do not hesitate to contact us if there are specific requests from the reviewers that you believe are technically impossible or unlikely to yield a meaningful outcome.

When revising your manuscript:

* Include a “Response to referees” document detailing, point-by-point, how you addressed each referee comment. If no action was taken to address a point, you must provide a compelling argument. This response will be sent back to the referees along with the revised manuscript.

* If you have not done so already please begin to revise your manuscript so that it conforms to our Article format instructions at http://www.nature.com/ni/authors/index.html. Refer also to any guidelines provided in this letter.

* Please include a revised version of any required reporting checklist. It will be available to referees to aid in their evaluation of the manuscript goes back for peer review. They are available here:

Reporting summary:
https://www.nature.com/documents/nr-reporting-summary.pdf

When submitting the revised version of your manuscript, please pay close attention to our href="https://www.nature.com/nature-research/editorial-policies/image-integrity">Digital Image Integrity Guidelines.</a> and to the following points below:

-- that unprocessed scans are clearly labelled and match the gels and western blots presented in figures.
-- that control panels for gels and western blots are appropriately described as loading on sample processing controls
-- all images in the paper are checked for duplication of panels and for splicing of gel lanes.

Finally, please ensure that you retain unprocessed data and metadata files after publication, ideally archiving data in perpetuity, as these may be requested during the peer review and production process or after publication if any issues arise.

Please use the link below to submit your revised manuscript and related files:
[REDACTED]

<strong>Note:</strong> This URL links to your confidential home page and associated information about manuscripts you may have submitted, or that you are reviewing for us. If you wish to forward this email to co-authors, please delete the link to your homepage.

We hope to receive your revised manuscript within 4 weeks. If you cannot send it within this time, please let us know. We will be happy to consider your revision so long as nothing similar has been accepted for publication at Nature Immunology or published elsewhere.

Please do not hesitate to contact me if you have any questions or would like to discuss these revisions further.

Nature Immunology is committed to improving transparency in authorship. As part of our efforts in this direction, we are now requesting that all authors identified as ‘corresponding author’ on published papers create and link their Open Researcher and Contributor Identifier (ORCID) with their account on the Manuscript Tracking System (MTS), prior to acceptance. ORCID helps the scientific community achieve unambiguous attribution of all scholarly contributions. You can create and link your ORCID from the home page of the MTS by clicking on ‘Modify my Springer Nature account’. For more information please visit please visit <a href="http://www.springernature.com/orcid">www.springernature.com/orcid</a>.

We look forward to seeing the revised manuscript and thank you for the opportunity to

review your work.

Sincerely,

Nicholas Bernard
Consulting Editor
Nature Immunology

Reviewers' Comments:

Reviewer #1:
Remarks to the Author:
Summary:
This study examines the humoral and cellular adaptive immune response to SARS-CoV-2 infection in children and adults, demonstrating that children are able to mount a more robust and more durable antibody and T cell response to infection. Importantly, the authors demonstrate that in children, the antibody response to SARS-CoV-2 infection appears to back-boost titers against seasonal beta-coronavirus strains and that this back-boosting is likely due to cross-reactivity with the S2 subunit among beta-coronavirus strains. This potential pre-existing immunity to SARS-CoV-2 is also evident with the T cell response, with both seropositive and seronegative patients (especially children) showing T cell responses to spike peptide mixes. Finally, the authors demonstrate that these more robust antibody and T cell responses in children enable them to maintain adaptive immunity at 6 months post-infection, demonstrating significant antibody titers against both the original Wuhan spike and VOCs for this length of time.

• I cannot easily interpret Figure 4C based upon the legend or main text results section. In the main text, the authors state:
"The proportion of responders against each pool was also different, with 98% of children with a positive ELISpot responding to spike but only 45% responding to the N/M pool. In adults, the proportions were 88% and 64%, respectively (Figure 4C)."
However, Figure 4C does not reflect these values as far as I can tell. I am confused how these calculations are made. This needs to be clarified either visually in the Figure or spelled out better in the text. The initial paragraph in that section (lines 244-246) sounds like the better interpretation of Figure 4C.
• It seems that the main message for Figure 6 is a bit veiled and pushed into the extended data table 3. At the end of the section, the authors state:

"Similar ratios of relative binding were seen in children and adults (Extended Data Table 3), demonstrating that improved recognition of VOC in children is a function of quantitively superior antibody responses."

Again, the way Figure 6 is drawn and discussed in most of the section needlessly confused that interpretation, giving the impression that antibody recognition in children was disproportionately superior at binding VOCs. The normalized data in the extended data table 3 of course shows it is actually a matter of overall titer levels in children at 6 months and not superior recognition of those VOCs in children per se. In other words, the authors do not show that children make antibodies with intrinsic superior recognition across VOCs, but that they make higher levels of antibodies and their titers are more durable than

adults. This is an important detail that should be addressed perhaps in more detail in the discussion. And, of course, the title of the manuscript is somewhat misleading in that regard.

• Overall, the authors have some very insightful results regarding the adaptive immune response to SARS-CoV-2 infection in children. The study cohort in particular is a very nicely controlled group of samples in which to compare adult and pediatric response to SARS-CoV-2 infection. For that reason in particular, I think these findings are high impact. However, the manuscript needs a bit of attention in how those results are described and interpreted, especially as noted for the above points. At times, I had to re-read sections several times to understand the relationship between the data/figures and what was being stated in the text.

Reviewer #2:
Remarks to the Author:
The study by Dowell et. al. and colleagues provides a fairly detailed comparative characterization of humoral and cellular immune profiles in seropositive cohorts of children and adults. The authors find non-significant but higher titers of IgG antibody against SARS-CoV-2 antigens (full length spike, RBD, N-protein) in seropositive children as compared to adults. When compared to seronegative children, seropositive children have broadly higher IgG titers against seasonal HCoVs which reaches significance for HKU1 and OC43. The boost in antibody titer is coronavirus specific as evidenced by absence of a similar response against influenza A and B and RSV. The S2 domain of spike but not S1 competed for binding to OC-43 and HKU1 in seropositive children. The authors probed the cellular immune response by peptide stimulation followed by ELISpot and multiplexed cytokine measurement assays. Intracellular staining was also performed on a small subset of pediatric samples. Interestingly the authors find a more robust T-cell response in seropositive children as compared to adults which is in contrast to data in a recent study (doi: https://doi.org/10.1101/2021.02.02.21250988). The antibody and cellular immune responses are maintained 6 months post infection in both adults and children but were more robust in the pediatric cohort with higher binding IgG titers against variants of concern (VOC) (B1.1.7, B1.351 and P1) as compared to seropositive adults.

This is a nice study yet there are some issues that prevent clear interpretation of the data. Please see below for a detailed explanation. In its current form, it may not be high priority for publication in Nature Immunology.

Major points:

1. A caveat to the experimental setup is absence of PCR test status against SARS-CoV-2 in the adults and children. Thus, true SARS-CoV-2 infection status was not known and "seropositivity" could be a consequence of CoV-2-cross reactive antibodies that were elicited by a seasonal CoV. This would be very interesting and a very different interpretation of the data. (If the study subjects were PCR+, this should be made very clear in the main text)

2. The authors have compared antibody titers between seropositive children and adults,

but disease severity of the seropositive subjects was not discussed. Given that antibody titers have been shown to correlate with disease severity in adults, a stratification of disease severity and comparison would help to interpret the data. If the adults and children had different types of infections (asymptomatic vs. symptomatic, mild vs. severe), this could be an alternative explanation for the differences in antibody responses.

3. In Figure 2, the authors have shown a boost in IgG titers against seasonal CoVs in seropositive children as compared to those who are seronegative yet a direct comparison between seropositive adults and children is missing. Thus, it is not clear that seropositive adults and children differ in the breadth of antibody responses against seasonal CoVs.

4. Though increased binding titers have been shown in children against VOCs as compared to adults (Figure 6), neutralization activity or other functional antibody data are not shown. Thus, it is not clear that there is any functional difference in the antibodies produced by children and adults.

5. A more detailed characterization of T-cell subsets and their associated cytokine signatures in adults vs children would add to the paper. The present data are suggestive of a cross-reactive T-cell response in sero-negative subjects, yet direct evidence is lacking.

Minor points:

1. It would be helpful to include the number of samples (n) tested in each figure legend.

2. The model figure is not very clear – may want to remove.

Reviewer #3:
Remarks to the Author:
The authors measured antibody levels and cellular immunity in children aged 3-11 years in comparison with adults. They report that children display high titers against Spike protein and receptor binding domain (RBD). SARS-CoV-2 seroconversion in children boosts antibody responses against seasonal beta-coronaviruses, partly through cross-recognition of the S2 domain. T cell responses against Spike are about two-fold higher in children compared to adults with Th1 cytokine profile. Children retains antibody titres and cellular responses for more than 6 months whereas antibody slightly wane in adults. At 6 months, in children the antibodies bound to various extent to Spike or RBD from B1.1.7, B1.351 and P1 variants. The study is well performed, confirming previous reports , but remains descriptive.
1. The study is based on a multiplex analysis of antibody levels (MSD technique). It would have been more informative to perform neutralization assays with pseudotyped virus or infectious virus to draw conclusions on antibody functions.
2. The authors often use in the terms “strong” or “high” to qualify theirs results. This is quite perturbing. For instance, Fig. 1 title: “children and adult induce strong coordinated antibody responses to SARS-CoV-2”. It is not surprising to see that SARS-CoV-2 infection triggers antibodies against different viral proteins. The word “Strong” appears in most of the figure titles.
3. Fig. 2. it seems that in SARS-CoV-2 seronegative individuals, antibody levels against seasonal coronavirus are higher in adults than in children. This may explain why the

authors show a “back-boosting” of the responses against seasonal HCoV upon SARS-CoV-2 infection more matrked in children than in adults.
4. Fig. 3. the authors state that antibody responses to S2 “contribute” to increased HCoV-specific antibody responses in SARS-CoV-2 seropositive children. However, they do not provide any formal demonstration of the role of these antibodies in this increase.
5. Fig. 4. The authors show higher Spike T cell responses in children than in adults with g-IFN Elispots. Measurement of cytokines does not show increased production of IL2 , IL4, IL10 and IL-17A. Could the authors confirm the Elispot by measuring increased secretion of g-IFN?
6. Antibody waning in adults at 6 months. There are multiple reports showing the half-life of anti-Spike antibodies is relatively long (>2 years). This should be further discussed. Is it possible that the sensibility of the MSD technique is lower than other serology techniques?

**Author Rebuttal to Initial comments**

**Response to Reviewers**

We would like to thank the Reviewers for their valuable comments and for the opportunity to revise our manuscript.

On the basis of this advice, we have undertaken a range of additional studies to enhance the manuscript. These comprise:

- Live virus and pseudotyped-virus neutralisation data, utilizing viral variants and including the delta variant
- Demonstration that SARS-CoV-2- specific T cells can be generated from seronegative children and thus demonstrating that cross reactivity against the spike protein extends to cellular response in addition to humoral immunity previously reported.
- Confirmation that this cross reactivity can also be shown using frozen blood samples from children that were obtained pre-pandemic and thus cannot have had any environmental exposure to SARS-CoV-2
- Assessment of the serological profile of children with a confirmed PCR-positive infection and demonstration that the antibody profile is the same as that seen in the seropositive convalescent cohort.
- Obtained blood samples from a subset of children at 12 months post infection and assessed the profile of antibody and cellular immunity. This shows broadly stable results compared to those at 6 months.

**Reviewers' Comments:**

**Reviewer #1:**

**Summary:**
This study examines the humoral and cellular adaptive immune response to SARS-CoV-2 infection in children and adults, demonstrating that children are able to mount a more robust and more durable antibody and T cell response to infection. Importantly, the authors demonstrate that in children, the antibody response to SARS-CoV-2 infection appears to back-boost titers against seasonal beta-coronavirus strains and that this back-boosting is likely due to cross-reactivity with the S2 subunit among beta-coronavirus strains. This potential pre-existing immunity to SARS-CoV-2 is also evident with the T cell response, with both seropositive and seronegative patients (especially children) showing T cell responses to spike peptide mixes. Finally, the authors demonstrate that these more robust antibody and T cell responses in children enable them to maintain adaptive immunity at 6 months post-infection, demonstrating significant antibody titers against both the original Wuhan spike and VOCs for this length of time.

*Thank you for these comments which summarize the manuscript perfectly.*

• I cannot easily interpret Figure 4C based upon the legend or main text results section. In the main text, the authors state:
"The proportion of responders against each pool was also different, with 98% of children with a positive ELISpot responding to spike but only 45% responding to the N/M pool. In adults, the proportions were 88% and 64%, respectively (Figure 4C)."
However, Figure 4C does not reflect these values as far as I can tell. I am confused how these calculations are made. This needs to be clarified either visually in the Figure or spelled out better in the text. The initial paragraph in that section (lines 244-246) sounds like the better interpretation of Figure 4C.

*Thank you. We apologise for not making this clear and the Figure and supporting text has now been completely revised.*

• It seems that the main message for Figure 6 is a bit veiled and pushed into the extended data table 3. At the end of the section, the authors state:

"Similar ratios of relative binding were seen in children and adults (Extended Data Table 3), demonstrating that improved recognition of VOC in children is a function of quantitively superior antibody responses."

Again, the way Figure 6 is drawn and discussed in most of the section needlessly confused that interpretation, giving the impression that antibody recognition in children was disproportionately superior at binding VOCs. The normalized data in the extended data table 3 of course shows it is actually a matter of overall titer levels in children at 6 months and not superior recognition of those VOCs in children per se. In other words, the authors do not show that children make antibodies with intrinsic superior recognition across VOCs, but that they make higher levels of antibodies and their titers are more durable than adults. This is an important detail that should be addressed perhaps in more detail in the discussion. And, of course, the title of the manuscript is somewhat misleading in that regard.

*Thank you. In retrospect we realise that this was not sufficiently clear and the text has been substantially revised to correct this. Moreover, we now have live virus and pseudotyped virus neutralisation data that has been included in the text. This also shows no qualitative superiority in the nature of the antibody response from children.*

• Overall, the authors have some very insightful results regarding the adaptive immune response to SARS-CoV-2 infection in children. The study cohort in particular is a very nicely controlled group of samples in which to compare adult and pediatric response to SARS-CoV-2 infection. For that reason in particular, I think these findings are high impact. However, the manuscript needs a bit of attention in how those results are described and interpreted, especially as noted for the above points. At times, I had to re-read sections several times to understand the relationship between the data/figures and what was being stated in the text.

*Thank you. We have made considerable textural changes to improve the clarity of the text. We feel that this has benefitted from the additional research findings discussed above.*

Reviewer #2:
Remarks to the Author:
The study by Dowell et. al. and colleagues provides a fairly detailed comparative characterization of humoral and cellular immune profiles in seropositive cohorts of children and adults. The authors find non-significant but higher titers of IgG antibody against SARS-CoV-2 antigens (full length spike, RBD, N-protein) in seropositive children as compared to adults. When compared to seronegative children, seropositive children have broadly higher IgG titers against seasonal HCoVs which reaches significance for HKU1 and OC43. The boost in antibody titer is coronavirus specific as evidenced by absence of a

similar response against influenza A and B and RSV. The S2 domain of spike but not S1 competed for binding to OC-43 and HKU1 in seropositive children. The authors probed the cellular immune response by peptide stimulation followed by ELISpot and multiplexed cytokine measurement assays. Intracellular staining was also performed on a small subset of pediatric samples. Interestingly the authors find a more robust T-cell response in seropositive children as compared to adults which is in contrast to data in a recent study (doi: https://doi.org/10.1101/2021.02.02.21250988). The antibody and cellular immune responses are maintained 6 months post infection in both adults and children but were more robust in the pediatric cohort with higher binding IgG titers against variants of concern (VOC) (B1.1.7, B1.351 and P1) as compared to seropositive adults.

This is a nice study yet there are some issues that prevent clear interpretation of the data. Please see below for a detailed explanation. In its current form, it may not be high priority for publication in Nature Immunology.

Major points:

1. A caveat to the experimental setup is absence of PCR test status against SARS-CoV-2 in the adults and children. Thus, true SARS-CoV-2 infection status was not known and "seropositivity" could be a consequence of CoV-2-cross reactive antibodies that were elicited by a seasonal CoV. This would be very interesting and a very different interpretation of the data. (If the study subjects were PCR+, this should be made very clear in the main text).

*Thank you. Yes, the main cohort was recruited as seropositive children and had not had a prior PCR test. As such this is a largely asymptomatic/mildly symptomatic paediatric cohort which is representative of the great majority of the childhood population.*

*The suggestion that 'seropositivity' is due to cross-reactive antibodies is an interesting one but we feel that this is unlikely for several reasons. Firstly, all the donors were seropositive for antibodies against spike and nucleocapsid and secondly these results were broadly stable over time. Cross reactive antibodies are generally much weaker and focussed against spike.*

*However, to address this question directly, we have also included analysis of a cohort of children with a prior positive PCR test. These confirm similar levels of SARS-CoV-2-specific antibody response to the cohort studied.*

2. The authors have compared antibody titers between seropositive children and adults, but disease severity of the seropositive subjects was not discussed. Given that antibody titers have been shown to correlate with disease severity in adults, a stratification of disease severity and comparison would help to interpret the data. If the adults and children had different types of infections (asymptomatic vs.

symptomatic, mild vs. severe), this could be an alternative explanation for the differences in antibody responses.

*Thank you. This is an interesting point. Both the children and adults had asymptomatic/mild infection with no hospital admission. We have added reference to this important point in the Discussion: "Antibody levels generally correlate with disease severity but none of the children or adults in this study had suffered from severe disease or needed hospital admission".*

3. In Figure 2, the authors have shown a boost in IgG titers against seasonal CoVs in seropositive children as compared to those who are seronegative yet a direct comparison between seropositive adults and children is missing. Thus, it is not clear that seropositive adults and children differ in the breadth of antibody responses against seasonal CoVs.

*Thank you. This is of note and indeed there is no difference in the HCoV-specific antibody titre between SARS-CoV-2 seropositive children and adults. Adults start with a somewhat higher level of such antibodies but this difference is 'filled' by SARS-CoV-2 infection. We have addressed this in the text. "Notably, the level of HCoV-specific antibodies in seropositive children was comparable to adults, whereas seronegative children possess lower responses than adults (Extended Data Table 1)*".

4. Though increased binding titers have been shown in children against VOCs as compared to adults (Figure 6), neutralization activity or other functional antibody data are not shown. Thus, it is not clear that there is any functional difference in the antibodies produced by children and adults.

*Thank you. This has now been completed, including assessment of the delta variant. This shows no difference in the functional capacity for neutralisation between children and adults.*

5. A more detailed characterization of T-cell subsets and their associated cytokine signatures in adults vs children would add to the paper. The present data are suggestive of a cross-reactive T-cell response in sero-negative subjects, yet direct evidence is lacking.

*Thank you. In order to address this we have expanded T cells from SARS-CoV-2 seronegative children, and frozen pre-pandemic blood samples, and confirmed that these demonstrate SARS-CoV-2 specific activity.*

Minor points:

1. It would be helpful to include the number of samples (n) tested in each figure legend.

*This has now been done*

2. The model figure is not very clear – may want to remove.

*Thank you. We have changed this on the basis of the additional data and we hope that this is now much more clear and informative.*

Reviewer #3:
Remarks to the Author:
The authors measured antibody levels and cellular immunity in children aged 3-11 years in comparison with adults. They report that children display high titers against Spike protein and receptor binding domain (RBD). SARS-CoV-2 seroconversion in children boosts antibody responses against seasonal beta-coronaviruses, partly through cross-recognition of the S2 domain. T cell responses against Spike are about two-fold higher in children compared to adults with Th1 cytokine profile. Children retains antibody titres and cellular responses for more than 6 months whereas antibody slightly wane in adults. At 6 months, in children the antibodies bound to various extent to Spike or RBD from B1.1.7, B1.351 and P1 variants. The study is well performed, confirming previous reports , but remains descriptive.
1. The study is based on a multiplex analysis of antibody levels (MSD technique). It would have been more informative to perform neutralization assays with pseudotyped virus or infectious virus to draw conclusions on antibody functions.

*Thank you. This has now been performed and has been very valuable.*

2. The authors often use in the terms “strong” or “high” to qualify theirs results. This is quite perturbing. For instance, Fig. 1 title: “children and adult induce strong coordinated antibody responses to SARS-CoV-2”. It is not surprising to see that SARS-CoV-2 infection triggers antibodies against different viral proteins. The word “Strong” appears in most of the figure titles.

*Thank you for this advice. It was very helpful to notice this repetition in our narrative and we have removed several references to 'strong' which now appears only once in the manuscript.*

3. Fig. 2. it seems that in SARS-CoV-2 seronegative individuals, antibody levels against seasonal coronavirus are higher in adults than in children. This may explain why the authors show a "back-boosting" of the responses against seasonal HCoV upon SARS-CoV-2 infection more marked in children than in adults.

*Thank you. We agree that we had not considered this sufficiently in the first draft and this has now been corrected (see above).*

4. Fig. 3. the authors state that antibody responses to S2 "contribute" to increased HCoV-specific antibody responses in SARS-CoV-2 seropositive children. However, they do not provide any formal demonstration of the role of these antibodies in this increase.

*Thank you. The use of spike S2 absorption shows that antibodies against the S2 domain represent a large component of the cross-reactive response. As the reviewer states, it is difficult to assess their relative functional role but, although these antibodies are unlikely to possess neutralising activity to the same extent as S1-specific antibodies, they may well have an important role mediated through complement fixation or Fc-mediated antibody dependent cytotoxicity (ADCC).*

5. Fig. 4. The authors show higher Spike T cell responses in children than in adults with g-IFN Elispots. Measurement of cytokines does not show increased production of IL2 , IL4, IL10 and IL-17A. Could the authors confirm the Elispot by measuring increased secretion of g-IFN?

*This is an interesting point but a major problem is that the IFN-$\gamma$ is retained on the ELISpot plate and is not available within the supernatant for cytokine assay.*

6. Antibody waning in adults at 6 months. There are multiple reports showing the half-life of anti-Spike antibodies is relatively long (>2 years). This should be further discussed. Is it possible that the sensibility of the MSD technique is lower than other serology techniques?

*Thank you. We agree that modelling of the trajectory of antibody waning after natural infection is difficult and uncertain at this point. This is difficult to determine now in adults as almost all have received Covid-19 vaccination. As advised, we have added comment on this within the Discussion.*

*"There is increasing confidence in the relative stability of SARS-CoV-2-specific memory B cell and antibody responses but studies of antibody waning after natural infection are now difficult to perform in adults due to the widespread adoption of Covid-19 vaccines".*

*The MSD technique has been utilised widely for SARS-CoV-2-specific analysis, and provides the platform for Operation Warp Speed, and so we do feel confident in this approach.*

**Decision Letter, first revision:**

**Subject:** Your manuscript, NI-A32053A

**Message:** Our ref: NI-A32053A

22nd Sep 2021

Dear Dr. Moss,

Thank you for your patience as we've prepared the guidelines for final submission of your Nature Immunology manuscript, "Children develop strong and sustained cross-reactive immune responses against Spike protein following SARS-CoV-2 infection, with enhanced recognition of variants of concern" (NI-A32053A). Please carefully follow the step-by-step instructions provided in the attached file, and add a response in each row of the table to indicate the changes that you have made. Please also check and comment on any additional marked-up edits we have proposed within the text. Ensuring that each point is addressed will help to ensure that your revised manuscript can be swiftly handed over to our production team.

We would like to start working on your revised paper, with all of the requested files and forms, as soon as possible (preferably within two weeks). Please get in contact with us if you anticipate delays.

When you upload your final materials, please include a point-by-point response to any remaining reviewer comments and please make sure to upload your checklist.

If you have not done so already, please alert us to any related manuscripts from your group that are under consideration or in press at other journals, or are being written up for submission to other journals (see: https://www.nature.com/nature-research/editorial-policies/plagiarism#policy-on-duplicate-publication for details).

In recognition of the time and expertise our reviewers provide to Nature Immunology's editorial process, we would like to formally acknowledge their contribution to the external peer review of your manuscript entitled "Children develop strong and sustained cross-reactive immune responses against Spike protein following SARS-CoV-2 infection, with enhanced recognition of variants of concern". For those reviewers who give their assent, we will be publishing their names alongside the published article.

Nature Immunology offers a Transparent Peer Review option for new original research manuscripts submitted after December 1st, 2019. As part of this initiative, we encourage our authors to support increased transparency into the peer review process by agreeing to have the reviewer comments, author rebuttal letters, and editorial decision letters published as a Supplementary item. When you submit your final files please clearly state in your cover letter whether or not you would like to participate in this initiative. Please note that failure to state your preference will result in delays in accepting your manuscript for publication.

<b>Cover suggestions</b>

As you prepare your final files we encourage you to consider whether you have any images or illustrations that may be appropriate for use on the cover of Nature Immunology.

Covers should be both aesthetically appealing and scientifically relevant, and should be supplied at the best quality available. Due to the prominence of these images, we do not generally select images featuring faces, children, text, graphs, schematic drawings, or collages on our covers.

We accept TIFF, JPEG, PNG or PSD file formats (a layered PSD file would be ideal), and the image should be at least 300ppi resolution (preferably 600-1200 ppi), in CMYK colour mode.

If your image is selected, we may also use it on the journal website as a banner image, and may need to make artistic alterations to fit our journal style.

Please submit your suggestions, clearly labeled, along with your final files. We'll be in touch if more information is needed.

Nature Immunology has now transitioned to a unified Rights Collection system which will allow our Author Services team to quickly and easily collect the rights and permissions required to publish your work. Approximately 10 days after your paper is formally accepted, you will receive an email in providing you with a link to complete the grant of rights. If your paper is eligible for Open Access, our Author Services team will also be in touch regarding any additional information that may be required to arrange payment for your article.

You will not receive your proofs until the publishing agreement has been received through our system.

Please note that <i>Nature Immunology</i> is a Transformative Journal (TJ). Authors may publish their research with us through the traditional subscription access route or make their paper immediately open access through payment of an article-processing charge (APC). Authors will not be required to make a final decision about access to their article until it has been accepted. <a href="https://www.springernature.com/gp/open-research/transformative-journals">Find out more about Transformative Journals</a>.

If you have any questions about costs, Open Access requirements, or our legal forms,

please contact ASJournals@springernature.com.

<B>Authors may need to take specific actions to achieve <a href="https://www.springernature.com/gp/open-research/funding/policy-compliance-faqs">compliance</a> with funder and institutional open access mandates.</b> For submissions from January 2021, if your research is supported by a funder that requires immediate open access (e.g. according to <a href=""https://www.springernature.com/gp/open-research/plan-s-compliance"">Plan S principles</a>) then you should select the gold OA route, and we will direct you to the compliant route where possible. For authors selecting the subscription publication route our standard licensing terms will need to be accepted, including our <a href=""https://www.springernature.com/gp/open-research/policies/journal-policies"">self-archiving policies</a>. Those standard licensing terms will supersede any other terms that the author or any third party may assert apply to any version of the manuscript.

Please use the following link for uploading these materials: [REDACTED]

If you have any further questions, please feel free to contact me.

Best regards,

Elle Morris
Senior Editorial Assistant
Nature Immunology
Phone: 212 726 9207
Fax: 212 696 9752
E-mail: immunology@us.nature.com

On behalf of

Nick Bernard, PhD
Senior Editor
Nature Immunology

Reviewer #1:
Remarks to the Author:
Summary of re-review: The original manuscript boasted a very impactful data set enabling a robust comparison of serological & cellular response to SARS-CoV-2 infection in children and adults. However, it initially lacked clarity in many of its interpretations. Upon re-review of the updated and revised manuscript, the authors have significantly improved the readability of their conclusions and backed them up with additional experiments/data.

Minor point: Figure 4C bottom left pie graph label appears to be misplaced

Reviewer #2:

Remarks to the Author:
I remain somewhat lukewarm on the significance of the findings, but the execution of the study is fine and the authors have adequately addressed our comments.

Reviewer #3:
Remarks to the Author:
The authors have addressed most of my concerns. the ms has been significanly improved. a minor comment. The first sentence of the abstract states that "SARS-CoV-2 infection is generally mild or asymptomatic in children but the biological basis for this is unclear. We studied..."
however, this is not really the question that was addressed here.

**Author Rebuttal, first revision:**

Thank you for the additional comments from the Reviewers.

We have now amended the minor point raised by Reviewer #1: “Minor point: Figure 4C bottom left pie graph label appears to be misplaced”

Reviewer #3 raised the following point: “A minor comment. The first sentence of the abstract states that "SARS-CoV-2 infection is generally mild or asymptomatic in children but the biological basis for this is unclear. We studied..." however, this is not really the question that was addressed here.” We note that the Editorial team have kept this first sentence in the proposed abstract and would concur with this decision. We have demonstrated pre-existing and cross-reactive humoral and T cell responses in children and as such would argue that, whilst we cannot directly provide conclusive evidence that these protect children from severe disease, we do feel that these data are indeed valuable for understanding the biological basis of their relative clinical protection.

**Final Decision Letter:**

**Subject:** Decision on Nature Immunology submission NI-A32053B
**Message:** In reply please quote: NI-A32053B

Dear Dr. Moss,

I am delighted to accept your manuscript entitled "Children develop robust and sustained cross-reactive spike-specific immune responses to SARS-CoV-2 infection" for publication in an upcoming issue of Nature Immunology.

The manuscript will now be copy-edited and prepared for the printer. Please check your calendar: if you will be unavailable to check the galley for some portion of the next month, we need the contact information of whom will be making corrections in your stead. When you receive your galleys, please examine them carefully to ensure that we have not inadvertently altered the sense of your text.

Acceptance is conditional on the data in the manuscript not being published elsewhere, or announced in the print or electronic media, until the embargo/publication date. These restrictions are not intended to deter you from presenting your data at academic meetings and conferences, but any enquiries from the media about papers not yet scheduled for publication should be referred to us.

Please note that <i>Nature Immunology</i> is a Transformative Journal (TJ). Authors may publish their research with us through the traditional subscription access route or make their paper immediately open access through payment of an article-processing charge (APC). Authors will not be required to make a final decision about access to their article until it has been accepted. <a href="https://www.springernature.com/gp/open-research/transformative-journals">Find out more about Transformative Journals</a>.

<B>Authors may need to take specific actions to achieve <a href="https://www.springernature.com/gp/open-research/funding/policy-compliance-faqs">compliance</a> with funder and institutional open access mandates.</b> For submissions from January 2021, if your research is supported by a funder that requires immediate open access (e.g. according to <a href="https://www.springernature.com/gp/open-research/plan-s-compliance">Plan S principles</a>) then you should select the gold OA route, and we will direct you to the compliant route where possible. For authors selecting the subscription publication route our standard licensing terms will need to be accepted, including our <a href="https://www.springernature.com/gp/open-research/policies/journal-policies">self-archiving policies</a>. Those standard licensing terms will supersede any other terms that the author or any third party may assert apply to any version of the manuscript.

In approximately 10 business days you will receive an email with a link to choose the appropriate publishing options for your paper and our Author Services team will be in touch regarding any additional information that may be required.

You will not receive your proofs until the publishing agreement has been received through our system.

If you have any questions about our publishing options, costs, Open Access requirements, or our legal forms, please contact ASJournals@springernature.com

Once your manuscript is typeset and you have completed the appropriate grant of rights, you will receive a link to your electronic proof via email with a request to make any corrections within 48 hours. If, when you receive your proof, you cannot meet this deadline, please inform us at rjsproduction@springernature.com immediately. Once your paper has been scheduled for online publication, the Nature press office will be in touch to confirm the details.

Your paper will be published online soon after we receive your corrections and will appear in print in the next available issue. Content is published online weekly on Mondays and Thursdays, and the embargo is set at 16:00 London time (GMT)/11:00 am US Eastern time (EST) on the day of publication. Now is the time to inform your Public Relations or Press Office about your paper, as they might be interested in promoting its publication. This will allow them time to prepare an accurate and satisfactory press release. Include your manuscript tracking number (NI-A32053B) and the name of the journal, which they

will need when they contact our office.

About one week before your paper is published online, we shall be distributing a press release to news organizations worldwide, which may very well include details of your work. We are happy for your institution or funding agency to prepare its own press release, but it must mention the embargo date and Nature Immunology. Our Press Office will contact you closer to the time of publication, but if you or your Press Office have any enquiries in the meantime, please contact press@nature.com.

Also, if you have any spectacular or outstanding figures or graphics associated with your manuscript - though not necessarily included with your submission - we'd be delighted to consider them as candidates for our cover. Simply send an electronic version (accompanied by a hard copy) to us with a possible cover caption enclosed.

To assist our authors in disseminating their research to the broader community, our SharedIt initiative provides you with a unique shareable link that will allow anyone (with or without a subscription) to read the published article. Recipients of the link with a subscription will also be able to download and print the PDF.

As soon as your article is published, you will receive an automated email with your shareable link.

You can now use a single sign-on for all your accounts, view the status of all your manuscript submissions and reviews, access usage statistics for your published articles and download a record of your refereeing activity for the Nature journals.

If you have not already done so, we strongly recommend that you upload the step-by-step protocols used in this manuscript to the Protocol Exchange. Protocol Exchange is an open online resource that allows researchers to share their detailed experimental know-how. All uploaded protocols are made freely available, assigned DOIs for ease of citation and fully searchable through nature.com. Protocols can be linked to any publications in which they are used and will be linked to from your article. You can also establish a dedicated page to collect all your lab Protocols. By uploading your Protocols to Protocol Exchange, you are enabling researchers to more readily reproduce or adapt the methodology you use, as well as increasing the visibility of your protocols and papers. Upload your Protocols at www.nature.com/protocolexchange/. Further information can be found at www.nature.com/protocolexchange/about .

Please note that we encourage the authors to self-archive their manuscript (the accepted version before copy editing) in their institutional repository, and in their funders' archives, six months after publication. Nature Research recognizes the efforts of funding bodies to increase access of the research they fund, and strongly encourages authors to participate in such efforts. For information about our editorial policy, including license agreement and author copyright, please visit www.nature.com/ni/about/ed_policies/index.html

An online order form for reprints of your paper is available at <a href="https://www.nature.com/reprints/author-reprints.html">https://www.nature.com/reprints/author-reprints.html</a>. Please let your coauthors and your institutions' public affairs office know that they are also welcome to order reprints by this method.

Sincerely,

Nick Bernard, PhD
Senior Editor
Nature Immunology
